# The impact of digital disability on the well-being of older adults: the moderating role of cultural deprivation

**DOI:** 10.3389/fpubh.2026.1713695

**Published:** 2026-02-19

**Authors:** LiChang Chen, Bing Xu, JinChan Li, YuJie Zhang, XinYi Jin, JingTong Li, LiXue Wang

**Affiliations:** 1 School of History and Culture, Zhengzhou Normal University, Zhengzhou, Henan, China; 2 School of History, Zhengzhou University, Zhengzhou, Henan, China

**Keywords:** aging, cultural deprivation, digital disability, moderation, subjective well-being

## Abstract

**Objective:**

This study aimed to investigate the impact of digital disability on the subjective well-being of older adult residents in Zhengzhou, with a specific focus on the moderating role of cultural deprivation in this relationship.

**Methods:**

An empirical research design was employed. Data were collected from a sample of older adult residents, and statistical analyses were conducted to examine the proposed relationships and moderation effect.

**Results:**

Digital disability (reverse-scored, with higher scores indicating greater competence) was significantly positively correlated with subjective well-being. Cultural deprivation significantly moderated this relationship. Simple slope analysis revealed that in contexts of higher cultural deprivation, digital disability exerted a more pronounced positive effect on well-being. Conversely, when deprivation levels were lower, this beneficial impact diminished significantly.

**Conclusion:**

Drawing on the law of diminishing marginal utility, this study elucidates the differentiated impact mechanisms of digital technology on older adults’ well-being. It underscores the importance of developing differentiated digital policies within a cultural welfare framework, thereby providing theoretical and practical insights for enhancing digital inclusion and well-being among the older population.

## 1 Introduction

As digitalization and aging converge, the issue of digital exclusion among the older adults has become increasingly prominent. Despite the Chinese government’s proactive efforts to address the digital divide among older adults through policies such as the Implementation Plan for Effectively Resolving Difficulties Older Adults Face in Using Smart Technologies, older adults continue to face a dual predicament of impaired daily functioning and cultural deprivation. This stems from declining physical capabilities, delayed technological adaptation, and barriers to digital access. According to the Statistical Report on China’s Internet Development, individuals aged 60 and above account for 20.8% of new internet users in China. However, only 41.2% of seniors can independently complete QR code payments, and their participation rate in digital cultural services remains below 15%. This aging society has given rise to a growing population of “digital refugees.” Exploring how digital exclusion impacts subjective well-being through cultural welfare is a key issue for achieving active aging.

Existing research has primarily focused on the mechanisms by which digital services influence the subjective well-being of older adults. Li notes that subjective well-being is a key indicator for measuring the quality of life in old age. Internet usage influences well-being not directly (1), but indirectly through mediators such as social connectedness and cultural participation (2–4). However, current research has largely focused on the psychological health dimension, with insufficient attention paid to the moderating role of cultural well-being. Cultural welfare, as a vital source of spiritual fulfillment, encompasses dimensions such as cultural services, activities, and education (5, 6). The extent to which individuals access such welfare may reshape the relationship between digital disability and well-being, though the underlying mechanisms warrant further investigation.

To clarify the above issues, this study takes the older adults in Zhengzhou as the research objects, and puts forward a conceptual framework with these three elements as the core by integrating the theoretical perspectives of digital disability, subjective well-being and cultural welfare deprivation. After defining the concepts in detail, this study infers that the reduction of the older adults digital disability will exert a positive impact on their subjective well-being. Moreover, this impact not only has a direct effect, but also produces an indirect effect by exacerbating their participation difficulties in the field of digital culture (cultural welfare deprivation). The theoretical framework aims to systematically reveal the complex path through which digital exclusion affects the psychological well-being of the older adults, verify the moderating effect of cultural welfare deprivation, and provide a clear blueprint for subsequent empirical tests.

This study, which targets older adult residents in Zhengzhou, has three primary objectives: first, to analyze the causal pathways through which digital disability impacts subjective well-being and examine the moderating role of cultural deprivation; second, to identify key determinants of digital disability and group differences; and third, to propose practical strategies for addressing digital disability and enhancing cultural welfare, thereby providing insights for advancing digital inclusion and cultural integration in an aging society.

## 2 Literature review

### 2.1 Digital disability: the dual dilemma of technological exclusion and social differentiation

Against the backdrop of accelerating digitalization, the older population faces increasingly prominent digital disability. According to the 53rd Statistical Report on China’s Internet Development, as of December 2023, the number of non-internet users aged 60 and above reached 126 million, accounting for 39.8% of the non-internet user population. This highlights the marginalization of older adults in the digital society. Digital disability, as an extension of the digital divide at the individual capability level, has become a core issue hindering the older population’s integration into the digital society (7). The academic community currently defines digital disability from multiple perspectives: Shan emphasizes, in a narrow sense, the inconvenience individuals experience in daily life due to insufficient capability, willingness, or need despite having access to digital resources. In a broader sense, it encompasses three-dimensional capability deficits: access gaps, usage gaps, and knowledge gaps (7). Zhang and Shan (8) defines it as “individuals’ maladaptive use and application barriers in digital scenarios,” further highlighting functional impairment among older adults in high-frequency application contexts. Wang (9) further proposes the concept of the “utility gap,” noting that older adults struggle to share in the economic, social, and cultural dividends of digitalization due to their inability to effectively utilize digital technologies, thereby becoming marginalized groups in the digital society.

In terms of measurement methodology, research has shifted from single-dimensional approaches to comprehensive assessments. Zhang and Shan (8) used “digital integration” as an entry point, employing a single indicator (frequency of online shopping) to measure the degree of digital disability among migrant workers; Shan and Zhang (7) developed a three-tiered framework inspired by physiological disability scales, categorizing digital disability into “complete lack of digital capability,” “partial deficiency,” and “relative deficiency”; Wang (10) proposed a novel measurement approach based on the third digital divide—“the utility gap”—advocating for evaluation standards centered on the actual empowerment effects of digital technologies on older adult lives.

Research trends are expanding in depth and breadth across multiple dimensions (11). In daily life, Octaviani et al. examined digital mobility, revealing that insufficient digital competence tangibly impede older adults’ transportation needs by undermining their ability and confidence to use digital ride-hailing services. In healthcare, Tharinee et al. (12) argue that digital and social exclusion heightens risks for medication adherence among seniors, making improved digital literacy crucial for medication safety. In cybersecurity, Pimpisa et al. (13) highlight that online fraud has become a significant factor affecting older adults’ digital usage and health, emphasizing the critical importance of enhancing their digital literacy and cultivating anti-fraud awareness.

Some studies reveal that urban–rural disparities exacerbate the complexity of digital disability. While urban seniors exhibit higher device ownership rates, they demonstrate insufficient proficiency in complex application scenarios, presenting a pattern of “high access but low utilization.” Rural populations, due to cognitive limitations and scarce recreational resources, are more susceptible to online addiction and fraud risks, creating a “digital divide” predicament (14). It is noteworthy that digital disability not only restricts the basic living needs of older adults but also significantly reduces their subjective well-being by diminishing social participation and positive perceptions (15). Although academia has proposed improving the current situation by alleviating digital disability, issues such as inconsistent measurement standards and divergent intervention approaches between urban and rural areas continue to constrain research depth. There is an urgent need to develop more explanatory theoretical frameworks and practical solutions.

Existing research has revealed the complex causes and negative impacts of digital disability among older adults from multiple perspectives, including conceptual definitions, measurement methods, analytical levels, and urban-rural disparities, providing a crucial foundation for understanding the digital divide. However, most studies remain confined to describing phenomena, lacking localized, multidimensional measurement tools for digital disability among China’s older adults. They particularly neglect empirical investigations into the “utility gap”—the actual empowerment effects of digital technologies. Furthermore, existing literature predominantly focuses on technological access and application capabilities, failing to delve into how digital disability impacts older adults’ psychological well-being through sociocultural pathways. This gap provides theoretical space for this study to construct a Digital Disability Scale and introduce cultural welfare variables.

### 2.2 Subjective well-being: the mediating mechanism and two-way path of digital influence

Well-being, as a philosophical topic spanning ancient and modern times, has been incorporated into the realm of quantitative science with the development of modern disciplines. Based on different philosophical traditions, Zhang (16) proposed that modern well-being research encompasses two models: one derived from subjectivism, focusing on subjective well-being, and the other evolved from the theory of fulfillment, emphasizing psychological well-being. Bradburn first proposed in 1969 that subjective well-being constitutes a psychological state formed by the balance of positive and negative emotions (17). Diener (18) further defined it as “an individual’s overall evaluation of their quality of life based on self-determined standards,” a concept widely accepted within academia. Early Chinese research on well-being primarily focused on older adults, with studies predominantly addressing mental health in later life. Li (1) emphasized that subjective well-being serves as a crucial indicator for measuring the quality of life in later adulthood.

Regarding influencing factors, Wang (19) categorizes the factors affecting subjective well-being into two levels: individual and societal. At the individual level, once older adults largely withdraw from the labor market, the lack of social connections and insufficient social support can lead to deteriorating mental health and the emergence of depressive symptoms (20). Furthermore, international research indicates that individuals with depression experience lower life satisfaction, resulting in diminished subjective well-being (21). Conversely, optimistic personality traits can positively enhance levels of well-being (22). At the societal level, well-being exhibits a near-U-shaped relationship with age, with the lowest levels observed among those aged 50–60 (23, 24). Regarding economic factors, it follows an inverted U-shaped curve: while income growth initially boosts well-being, the marginal effect diminishes as income increases (25).

Today, the impact of digitalization on subjective well-being exhibits a double-edged sword effect: on one hand, online social interactions and participation in cultural activities can increase social opportunities and communication platforms, strengthen emotional connections with modern society, and reduce negative emotions (26, 27). Tabet (28) also found that when older adults engage in socializing, entertainment, and other activities online to fulfill their needs, their subjective well-being improves. On the other hand, excessive online engagement may weaken real-world interpersonal relationships and diminish life satisfaction (29).

Extensive research indicates that mediating variables exist in the mechanism of well-being among older adults. Yang (30), through a comprehensive literature review, concluded that internet use among older adults typically does not directly influence subjective well-being but rather indirectly affects it through other factors, suggesting the presence of mediating or moderating effects. Internet use indirectly enhances subjective well-being via mediating pathways such as strengthening social support, intergenerational support, and boosting self-esteem (1, 31). Vega et al. introduced the Self-Efficacy Scale, concluding that the strength of the relationship between digital disability and well-being is moderated by self-efficacy, thereby broadening the research perspective in this field (59).

However, the impact of digital disability on subjective well-being exhibits group heterogeneity: rural older adults with low digital disability have significantly improved their well-being through online social networking and entertainment (32), In contrast, the group with high digital disability faces digital ageism due to high risks of online fraud and intense technological anxiety (33).

Existing research has clarified the multidimensional structure of subjective well-being and its significance among older adults, progressively revealing the mediating pathways through which digitalization influences well-being and its double-edged sword effect. However, most studies adopt a singular psychological or sociological perspective, failing to integrate a comprehensive “technology-culture-psychology” analytical framework. Notably absent is an exploration of the mechanisms through which cultural resources mediate the relationship between digital technology and well-being. Most studies remain confined to single indicators such as internet usage frequency, failing to distinguish between different dimensions of digital disability and their interactions with cultural welfare. This limitation hinders a deeper understanding of pathways to enhance well-being among older adults and justifies introducing cultural deprivation as a moderating variable in this research.

### 2.3 Cultural well-being: paradigm shift from physical provision to digital empowerment

In the digitalization process, most scholars have recognized the importance of “cultural welfare” development for people’s sense of well-being. They believe cultural activities serve as one of the key ways for older adults to strengthen their self-connection and social connections. Appropriate social participation can reduce feelings of loneliness among older adults and alleviate potential anxiety arising from social disengagement (34–36).

The definition of cultural welfare has expanded alongside digitalization, yet academic consensus remains elusive. Two primary perspectives exist: first, viewing it as a component of social welfare based on the welfare concept, emphasizing government-led public cultural service provision and a multi-stakeholder welfare supply system dominated by government with societal support (37–39). The second perspective integrates digital trends by proposing the concept of “public digital cultural services,” advocating for expanding cultural resource accessibility through information technology (40).

Extensive research findings reveal imbalances in urban-rural supply: rural areas face challenges such as the formalization of facility construction and a disconnect between content and demand (39, 41), with farmers’ latent demand for cultural activities remaining underutilized (42). Meanwhile, urban communities suffer from a mismatch between cultural service supply and demand due to the lack of constructive interaction among the government, community organizations, and residents (43).

It is worth pondering that as the pace of digitalization accelerates and cultural welfare increases, the older adults face heightened barriers to accessing cultural welfare due to digital exclusion. Concurrently, research indicates that high-quality cultural participation—such as offline community opera classes and senior choirs—can significantly enhance older adults’ social integration and life satisfaction (44). Digital cultural production alleviates negative emotions, fulfills psychological needs, and enhances well-being by providing entertainment and leisure opportunities for older adults. This suggests that cultural welfare may mitigate the negative impacts of digital disability through two pathways: offline cultural activities provide alternative channels for participation, while online digital cultural services alleviate technological anxiety. However, existing research has yet to systematically validate the moderating effect of cultural welfare, particularly lacking in-depth exploration of the enabling mechanisms of digital cultural services. The enhancement of older adults’ subjective well-being through participation in online cultural activities, while improving their digital literacy and cultural engagement, remains a pressing challenge.

In summary, existing research has generally focused on the provision and value of cultural welfare, but it has largely been confined to theoretical discussions and descriptive rather than explanatory analyses. There has been a lack of empirical testing of its moderating effects, particularly failing to reveal the specific mechanisms through which cultural welfare shapes the relationship between digital disability and well-being. This gap thus provides a research opportunity for this study to develop a cultural deprivation measurement scale and validate the moderating effects of cultural deprivation.

### 2.4 Literature review

Through a systematic review of relevant literature, we have clarified the current state of research on digital disability and subjective well-being among older adults, as well as the relationships among digital disability, cultural welfare, and subjective well-being in this population. However, we find that while existing research has made significant progress in the fields of digital disability, subjective well-being, and cultural welfare, deficiencies remain at the theoretical, methodological, and policy levels:

Theoretically, the interactive mechanisms among these three elements remain unclear. Digital disability research primarily focuses on technological exclusion, analyses of subjective well-being emphasize psychological mechanisms, and discussions on cultural welfare are limited to supply models, with few collaborative studies integrating all three. As a potential moderating variable, the pathways through which cultural welfare mitigates the negative effects of digital disability have not been systematically validated, particularly lacking empirical exploration of how digital cultural services influence subjective well-being. This study employs quantitative research methods, treating cultural welfare as a moderator to investigate its role in the relationship between digital disability and subjective well-being.

Currently, no validated and comprehensive scales for digital disability or cultural deprivation have been developed globally. Measurements of digital disability remain limited to single dimensions or metrics, overlooking physiological and cognitive characteristics such as delayed touchscreen response times and comprehension barriers among older adults. This approach fails to fully capture the true extent of digital disability within the older adult population. Measurements of cultural welfare predominantly rely on traditional hardware coverage metrics, lacking designs that assess the penetration and depth of participation in digital cultural services. Although existing research has attempted to measure digital disability and cultural welfare, no universally recognized, specialized scales tailored for China’s older adult population currently exist. Therefore, this study preliminarily designed scales for measuring digital disability and cultural deprivation among the older adults through literature review, preliminary research, and expert consultation.

In terms of countermeasures, the recommendations for addressing these three issues lack organic integration. Through examining the relationships among them, this study proposes scientifically sound and reasonable countermeasures.

This study focuses on the older population in Zhengzhou, achieving breakthroughs through triple innovation in theory, tools, and policy recommendations: (i) Theoretical innovation: Constructing a “technology-culture-psychology” synergistic framework and proposing the core hypothesis that cultural welfare serves as a moderating variable to mitigate the negative effects of digital disability. (ii) Tool innovation: Developing scales tailored for the older adults to measure digital disability and cultural welfare deprivation. (iii) Policy innovation: Integrating these three dimensions to formulate targeted recommendations. The findings are expected to enrich theories on the moderating role of cultural welfare, provide scientific grounds for government policies on digital aging-friendliness, and ultimately facilitate the transition of the older adult population from “technological exclusion” to “digital empowerment.” This approach aims to achieve dual objectives in the aging era: “digital empowerment” and “cultural enrichment.”

## 3 Theoretical analysis and research hypotheses

### 3.1 The relationship among digital disability, cultural welfare, and well-being

Research indicates that traditional media formats such as newspapers, television, and radio can effectively enhance older adults’ sense of social participation (45). Simultaneously, the internet, as an emerging cultural form, further expands the breadth and depth of older adults’ social engagement. However, the “digital disability” faced by some older adults in adapting to the digital environment may limit their effective access to and utilization of digital cultural resources, thereby affecting their psychological experiences and subjective perceptions. Cultural well-being, as a core indicator reflecting an individual’s quality of cultural life, together with digital competence, constitutes an important factor influencing older adults’ subjective well-being (46).

To delve into the intrinsic logic among these three elements, this study introduces Maslow’s hierarchy of needs theory as an integrative framework. Maslow’s Hierarchy of Needs categorizes human needs into five levels, arranged from lowest to highest: physiological needs, safety needs, social needs, esteem needs, and self-actualization needs. This theory posits that human needs begin with basic physiological needs, gradually progressing toward fulfillment until reaching the highest level: the satisfaction of self-actualization needs. For older adults, higher-level emotional needs, such as social belonging, respect, and self-actualization, are particularly crucial (47). From this perspective, access to cultural welfare can be regarded as a key pathway to fulfilling the higher-level spiritual needs of the older adults, such as social media usage influences older adults’ subjective well-being through two dimensions: positive emotions and life satisfaction (48). As social media use constitutes both a cultural welfare and a vital channel for older adults to engage with cultural activities, digital literacy is intrinsically linked to cultural welfare. Therefore, digital literacy contributes to fulfilling older adults’ higher-level psychological needs by facilitating access to and engagement with cultural benefits, thereby influencing their subjective well-being (49).

In summary, there is a close intrinsic connection among digital disability, cultural welfare deprivation and subjective well-being. It should be noted that both the “digital disability” and “cultural welfare deprivation” variables in this study are reverse-scored, meaning that a higher score on the scale indicates a lower level of actual disability or deprivation. Based on this, the following hypotheses are proposed:

*Hypothesis 1:* The subjective well-being of the older adult is positively correlated with cultural welfare deprivation (a higher score on the cultural welfare deprivation scale indicates a higher level of subjective well-being).

*Hypothesis 2:* The digital disability of the older adult is positively correlated with their subjective well-being (a higher score on the Digital Disability Scale indicates a higher level of subjective well-being).

*Hypothesis 3:* There is a significant correlation among the digital disability, cultural welfare deprivation and subjective well-being of the older adult(s).

### 3.2 The mechanism of cultural welfare for the older adults

Research indicates that enabling the older adult to access shared public digital cultural services can broaden their avenues for cultural access, safeguard their cultural rights, thereby overcoming digital disability, fulfilling their sense of self-worth, and enhancing their subjective well-being (50). Digital public cultural services also constitute part of cultural welfare, offering insight into the current state of cultural participation among older adults to a certain extent. Zhang et al. (47) observed a growing demand for digital reading among older adults, with increased digital reading correspondingly driving higher levels of cultural engagement. Promoting digital reading can bridge the digital divide for older adults by providing essential information relevant to key aspects of their lives. This enhances their information literacy, enriches their later years, and increases their sense of well-being. Xin and Chen (51) argue from a short-video perspective that such videos not only enhance older adults’ digital skills but also alleviate feelings of alienation, marginalization, and distance within family and social settings. This, to some extent, promotes social integration among the older adults, thereby boosting their sense of well-being and belonging. These findings reflect the positive moderating role of cultural welfare in the relationship between digital disability and subjective well-being.

Additionally, some scholars have pointed out that excessive cultural welfare can have negative consequences. While the use of online media can provide solace through interaction, this form of vicarious comfort may deepen older adults’ dependence on and addiction to the internet. This, in turn, can adversely affect their physical and mental health and diminish their subjective well-being (52). Beyond this, excessive participation in online cultural activities can crowd out offline cultural engagement for the older adults, thereby reducing interaction with family members, particularly their children, and diminishing their sense of well-being. More concerning is the tendency at the grassroots level to prioritize meeting metrics for digital learning and activities among seniors without considering their genuine needs, resulting in a form of “digital formalism.” This detrimental approach to cultural activities not only fosters resistance to learning digital technologies among the older adults but also undermines their overall well-being (53).

According to the law of diminishing marginal utility in economics, the differential impact of digital disability can be deduced. For older adults experiencing high levels of cultural deprivation, digital technology serves as a scarce substitute resource with exceptionally high marginal utility. Learning to use WeChat video calls may signify a profound emotional connection with distant children, while mastering QR code payments could represent a significant leap toward shopping autonomy. Consequently, therefore, the reduction of digital disability level will lead to a significant improvement in the level of subjective well-being. Conversely, for seniors with ample cultural welfare provision, their social, recreational, and informational needs are already well-met through offline activities (such as senior universities and community centers). In this context, digital technology functions as a supplementary tool, exhibiting diminishing marginal utility. The satisfaction derived from learning a new app feature pales in comparison to that of the previously mentioned cases. Moreover, excessive use of digital devices may crowd out participation in high-quality offline activities, thereby diminishing their positive effects. Therefore, we predict that the impact intensity of digital disability is not fixed, but rather weakens as the level of cultural welfare deprivation increases.

*Hypothesis 4:* Cultural welfare deprivation moderates the relationship between digital disability (reverse-scored) and subjective well-being. Specifically, when the level of cultural welfare deprivation is high, a higher score on the Digital Disability Scale (stronger digital competence) exerts a positive effect on improving well-being; whereas when the level of cultural welfare deprivation is low, this positive effect diminishes.

## 4 Data sources and measurement

### 4.1 Data sources

The study was conducted in Zhengzhou City, targeting older adults aged 50 and above. Fieldwork involved visiting 6 communities, 4 villages, 2 parks, and 3 nursing homes. A total of 200 online and offline questionnaires were distributed, with 182 valid responses collected.

### 4.2 Scale design and validation

#### 4.2.1 Independent variables

##### 4.2.1.1 Scale development

To ensure the innovation of the research and the validity of the instrument, the compilation process strictly follows psychometric standards (54). This study attempts to establish indicators for digital disability among the older adults. It adopts the three primary indicators proposed by Wang (10), namely digital access, digital competence, and digital usage. Based on the field research conducted in Zhengzhou, a digital disability measurement framework consisting of 3 dimensions and 9 secondary indicators has been initially constructed. The indicators cover three levels: access, skills, and usage, aiming to comprehensively reflect the digital dilemmas faced by older adults. During the development process, preliminary indicators were first constructed based on the theoretical framework. Five experts in relevant fields were invited to evaluate the indicator weights using the Analytic Hierarchy Process (AHP). The consistency ratio (CR) of all judgment matrices was less than 0.1, ensuring content validity. Following pretesting, items were validated through item analysis and exploratory factor analysis. Evaluation criteria included factor loadings (>0.5), cross-loadings, and theoretical consistency. All 27 items met retention standards, with no items removed. The formal analysis revealed a KMO value of 0.933 for the scale, with Bartlett’s Sphericity test showing significant results (*χ*^2^ = 5898.379, *p* < 0.001). Principal component analysis extracted five common factors with eigenvalues greater than 1. After rotation, the factor structure aligned well with the theoretical dimensions, indicating ideal construct validity. The overall Cronbach’s *α* coefficient for the scale was 0.975, with all dimensions meeting reliability standards, indicating extremely high internal consistency.

##### 4.2.1.2 Variable measurement

This study used a self-developed Digital Disability Scale for measurement. The scale consists of 27 items, with a total score ranging from 27 to 135, as shown in Table 1. A 5-point Likert scale is employed, where higher scores indicate lower levels of digital disability among the older adults and greater understanding and application of the internet. The total score range is 27 to 135 points. A total score <70 indicates severe digital disability; 70 ≤ total score ≤ 100 indicates moderate digital disability; and a total score >100 indicates mild digital disability.

**Table 1:** Measures of digital disability.

Level of digital disability	Corresponding indicators	Corresponding questions (variables)
Digital access	Device accessibility	I have a smartphone that can access the internet and functions normally.
		I can afford the monthly phone bill and data plan.
	Network coverage	My phone’s network is strong, allowing smooth browsing of news and watching videos.
		My data plan and usage time fully meet my daily needs.
Digital skills	Basic operational skills (physical contact and simple interaction)	I can independently use my smartphone to make and receive calls.
		I am proficient in sending and receiving text messages or WeChat voice messages.
		I can adjust basic settings like volume and brightness.
	Communication skills	I can participate in online group chats (community activity groups) via my phone.
		I can use the internet to communicate with others—chatting with family on WeChat, sharing videos with friends on Douyin.
		I can proficiently take photos and post them on Moments or Douyin.
	Information security and privacy protection skills	I can set privacy protections like phone passwords to safeguard personal information.
		I can recognize scam calls and texts and refuse to answer them.
		I can protect my finances online—verifying recipient details when using mobile payments.
		I can search for information and stay informed about current events via mobile internet platforms.
	Problem-solving skills	When my phone lags, I clear memory and restart it to restore performance.
		I resolve technical and information issues by calling customer service—e.g., recharging credits, checking data usage.
		I learn new features by following manuals—e.g., QR code payments.
	Information interaction skills	I complete QR code payments using WeChat/Alipay.
		I book hospital appointments and make online payments via mobile apps.
		I plan travel routes using map navigation software.
		I proficiently use public services apps to pay utilities, social insurance, and check pension balances.
		I can search for products, place orders, and process returns/exchanges on e-commerce platforms (e.g., Taobao).
Digital usage	Frequency of use	I use my phone for over an hour daily (scrolling through Douyin, chatting, reading news).
		I frequently use my phone and other digital devices weekly.
	Versatility in usage scenarios	I’ve used my phone for: shopping, government services, and social networking.
		I often use my phone to listen to music, watch operas, tune into the radio, and read novels.
		I regularly use my phone for learning and acquiring new skills.

#### 4.2.2 Dependent variable

##### 4.2.2.1 Scale development

Subjective well-being was measured using the Memorial University of Newfoundland Subjective Well-Being Scale (55). Developed by Kozma and Stones in 1980, the MUNSH is a well-being assessment tool widely used in gerontological research, comprising four subscales: Positive affect (PA), Negative affect (NA), Positive experience (PE), and Negative experience (NE), with a total of 24 items. To meet the needs of Chinese localization and improve the precision and discriminability of the scale, Zhu (56) expanded the original 3-point scale to a 5-point scale, as shown in Table 2. This Chinese version of the scale has demonstrated good psychometric properties among the older adult population in China. For instance, a study by Ren (57) reported a Cronbach’s *α* coefficient of 0.832, providing a comparable reference standard for the results of this study. In the present study, the test of construct validity showed that the KMO value was 0.822, and the Bartlett’s test of Sphericity was significant (*χ*^2^ = 2023.061, *p* < 0.001). The overall Cronbach’s *α* coefficient of the scale was 0.797, with coefficients for each sub-dimension greater than 0.7, indicating good reliability and suitability for this study.

**Table 2:** Methods for measuring subjective well-being.

Level of subjective well-being	Corresponding questions (variables)
Positive affect (PA)	Extremely satisfied?
	In a very good mood
	Very satisfied with your life?
	Very fortunate
	Generally speaking, have your life circumstances become more satisfying?
Negative affect (NA)	Worries
	Feeling very lonely or isolated
	Feeling depressed or very unhappy
	Worried about what the future holds
	Feeling your life circumstances have become difficult
Positive experience (PE)	I feel as happy as I did when I was young
	The things I do interest me as much as they used to
	Looking back on my life, I feel quite satisfied
	If you could live anywhere you wanted, where would you choose to live?
	Am I as happy now as I was when I was young?
	Were you satisfied with your life back then?
	My health is the same as or even better than that of my peers
Negative experience (NE)	This is the most difficult period of my life
	Most of what I do is tedious or monotonous
	As I get older, everything gets worse.
	Do you feel lonely?
	Have some things troubled me this year?
	Sometimes I feel life is meaningless.
	Most of the time I feel life is hard

##### 4.2.2.2 Variable measurement

Total scores are calculated as follows: PA + PE − NA − NE. For statistical convenience, the total score was adjusted to range from 0 to 96 by adding 48. Scores below 50 indicate low emotional factors, 50 ≤ total score ≤ 80 indicate moderate emotional factors, and 80 < total score ≤ 100 indicate high emotional factors.

#### 4.2.3 Moderating variables

##### 4.2.3.1 Scale development

Given the lack of consensus within the academic community regarding the precise definition of cultural welfare, related research remains fragmented, and no established measurement tools have yet emerged. This study primarily conducts a preliminary classification of cultural welfare research indicators through a review of scholarly literature by Li (5) and Che (58), as well as others, and an interpretation of policies, including the Zhengzhou Municipal “14th Five-Year Plan” for cultural, radio, television, and tourism development. The primary indicator is subdivided into four dimensions: participation in online cultural services, access to online cultural information, barriers to offline smart services, and perception of cultural benefits. This scale was initially developed based on a theoretical framework and comprises 21 items. First, five community cultural service experts were invited to evaluate the dimensions using the AHP method. The experts demonstrated good agreement (CR < 0.1), ensuring content validity. Following pretesting, item refinement was conducted through item analysis and exploratory factor analysis. Based on criteria including factor loadings (>0.5), cross-loadings, and theoretical fit, two items with marginal statistical performance and inconsistent alignment with the predefined dimensions were removed, resulting in 19 retained items. The formal analysis revealed a KMO value of 0.916 and a significant Bartlett’s Sphericity test (*χ*^2^ = 2576.473, *p* < 0.001). The extracted four common factors aligned with the theoretical dimensions, indicating good construct validity. The overall Cronbach’s *α* coefficient for the scale was 0.934, with all dimensions meeting reliability standards, demonstrating good internal consistency.

##### 4.2.3.2 Variable measurement

These comprise 19 items in total, all scored using a 5-point Likert scale, as shown in Table 3. Higher scores indicate lower levels of cultural deprivation and greater cultural fulfillment among the older adults. The total score range is 19 to 95. A total score <45 indicates severe cultural deprivation; 65 ≥ total score ≥ 45 indicates moderate cultural deprivation; and a total score >65 indicates mild cultural deprivation.

**Table 3:** Measurement methods for cultural deprivation.

Level of cultural deprivation	Corresponding questions (variables)
Online cultural deprivation	Video category: I frequently watch short videos, opera performances, square dances, etc., on platforms like Douyin and Kuaishou.
	Video category: I can easily find long-form videos, TV series, and movies that interest me on Tencent Video/iQIYI/Youku/Bilibili.
	Video category: I often watch live events such as talent shows and intangible cultural heritage performances.
	Audio category: I regularly listen to audiobooks, radio dramas, and storytelling programs, such as those on Himalaya.
	Reading category: I frequently read e-books/digital novels
Access to online cultural information	Knowledge popularization category: I acquire health/wellness knowledge, historical/cultural knowledge, and scientific knowledge through online lectures/open courses
	Knowledge popularization category: I receive updates on intangible cultural heritage events and festival information via official websites/government portals/Ministry of Culture and Tourism/local cloud platforms
	Art appreciation category: I appreciate cultural relics and landscapes through virtual exhibitions (museum cloud platforms, online Dunhuang tours, VR platforms)
	Art appreciation: I watch online performances via the National Centre for the Performing Arts’ “Cloud Theater” or live streams from local opera troupes
	Practical skills: I learn paper-cutting, weaving, and cooking tutorials through platforms like Douyin and Kuaishou
	Practical skills: I access illustrated guides for “mobile registration, ride-hailing, and advance appointments” via community official accounts, mini-programs, or Xiaohongshu
Barriers to offline smart services	Venue reservations & guided tours: I can independently complete online reservations for museums/libraries without assistance from children or volunteers
	Venue reservations & guided tours: I can independently operate AR maps at the Forbidden City and immersive exhibition equipment at memorial halls without staff guidance
	Self-service facilities: I can independently use library self-checkout systems via facial recognition or QR code scanning
	Self-service facilities: I can independently rent and return self-guided audio devices
	Community cultural activities: I can independently register for community events via WeChat groups/mini-programs without needing children to set reminders
	Community cultural activities: I can independently accumulate points for offline cultural activities via apps and redeem gifts
Perception of cultural welfare	Participating in cultural activities brings me joy
	I’ve made new friends through cultural activities
	Current cultural resources meet my spiritual needs
	Smart services have enhanced the convenience of my cultural participation

## 5 Empirical analysis

### 5.1 Descriptive analysis

Tables 4, 5 presents the descriptive analysis results for the variables selected in this study.

**Table 4:** Descriptive statistical analysis of control variables (*N* = 182).

Variable name (type)	Category	Frequency (*N*)	Percentage (%)
Gender	1 = Male	85	46.70
	2 = Female	97	53.30
Age	1 = 50–55 years old	54	29.67
	2 = 56–60 years old	32	17.58
	3 = 61–65 years old	28	15.38
	4 = 66–70 years old	34	18.68
	5 = 71–75 years old	26	14.29
	6 = Over 76 years old	8	4.4
Education level	1 = no formal education	14	7.69
	2 = literate but no formal education	4	2.2
	3 = elementary school	58	31.87
	4 = junior high school	62	34.07
	5 = high school/vocational school	36	19.78
	6 = college or higher	8	4.4
Residence	1 = rural areas	115	63.19
	2 = urban communities	60	32.97
	3 = migrant populations (accompanying children or working)	7	3.85
Source of income	1 = pension	15	8.24
	2 = support from children	36	19.78
	3 = government subsidy	8	4.4
	4 = labor income	113	62.09
	5 = other	10	5.49
Health status	1 = fully self-care	162	89.01
	3 = partially in need of assistance	20	10.99
Relationship with children	1 = intimate	160	87.91
	2 = neutral	22	12.09
Occupation	1 = farmer	102	56.04
	2 = individual/self-employed business owner	30	16.48
	3 = worker/commercial service staff	26	14.29
	4 = government/party and government institution staff	2	1.1
	5 = science, technology, medical and health worker	8	4.4
	6 = cultural and educational worker	14	7.69
	7 = other	102	56.04

**Table 5:** Descriptive statistical analysis of continuous variables (*N* = 182).

Variable	Minimum	Maximum	Mean	Standard deviation
Dependent variable				
Subjective well-being	36	88	62.28	10.629
Independent variable				
Digital disability	59	125	91.214	14.013
Moderating variable				
Cultural deprivation	28	80	54.154	10.277

#### 5.1.1 Variable distribution characteristics

The mean subjective well-being score was 62.28, indicating that overall Well-being among the older adult population was at a moderate level. However, significant individual variation was observed, with the highest score exceeding the lowest by 52 points. The mean score for digital disability (reverse-scored scale) was 91.21, reflecting intense differentiation within the group. Some older adults exhibited mild digital disability, scoring near the maximum, while the lowest score was only 59, exposing severe disparities in digital disability levels. The mean score for cultural deprivation (reverse-scored) was 54.15, indicating uneven cultural participation. While some older adults enjoyed ample access to cultural welfare, those with low scores faced substantial cultural deprivation.

#### 5.1.2 Sample structural characteristics

The age distribution shows a mean of 2.835, with the sample concentrated in the 50–65 age group (accounting for approximately 60%). The proportion of people aged 65 and above is relatively low. The primary residence is predominantly rural, and farmers constitute the largest occupational group. The educational attainment of the sample is mostly concentrated in primary school and junior high school, and their income is mainly derived from self-employment labor. This indicates that the sample as a whole has a low-to-moderate socioeconomic status, limited cognitive ability, and restricted access to cultural and recreational resources.

### 5.2 Analysis of variance

Table 6 reveals significant differences in digital competence and cultural welfare across age groups. The digital adaptability of the older adult shows a marked decline with increasing age (*F* = 14.826, *p* < 0.001). The 50–55 age cohort scored highest, likely due to active digital demands in the workplace. However, scores plummeted sharply after age 61, reflecting dual constraints: reduced technological needs post-retirement and physiological decline. Cultural welfare access exhibits dynamic fluctuations. Although subjective well-being showed no significant age differences (*F* = 0.231, *p* = 0.949), it revealed underlying structural contradictions: the 50–55 age group with the strongest digital adaptation did not exhibit significantly higher well-being than other groups, suggesting the paradox that “Technological empowerment does not necessarily translate to well-being gains” Meanwhile, the 76+ age group exhibited highly volatile well-being scores (±15.55), suggesting that individual differences in advanced age may be influenced by non-technological factors such as health and family circumstances.

**Table 6:** Comparison of scale scores across different age groups (mean ± standard deviation).

Age	Sample size	Digital Disability Scale	Cultural welfare deprivation scale	Subjective Well-Being Scale
50–55	54	99.91 ± 10.40	54.78 ± 8.43	61.76 ± 11.71
56–60	32	95.34 ± 11.28	59.78 ± 9.23	63.63 ± 8.63
61–65	28	87.25 ± 16.02	50.75 ± 11.75	62.54 ± 9.68
66–70	34	89.32 ± 11.14	54.26 ± 8.79	62.82 ± 11.51
71–75	26	78.65 ± 9.98	49.50 ± 11.11	61.15 ± 9.28
76 above	8	78.75 ± 16.08	54.00 ± 14.28	60.88 ± 15.55
*F*		14.826	3.936	0.231
*p*		0.000**	0.002**	0.949

Table 7 reveals that educational attainment exhibits significant differences in relation to digital competence, cultural welfare, and subjective well-being. Comparing these means and standard deviations indicates a positive correlation between educational attainment and each variable. Digital adaptability exhibits a nonlinear correlation with educational attainment (*F* = 3.798, *p* = 0.003): Junior high school graduates scored the highest, even surpassing those with college degrees or higher. This may stem from their occupational contexts, which drive the forced adoption of technology; for instance, individual merchants frequently use mobile payments and order-taking platforms. The literate but uneducated group demonstrated notably strong performance, reflecting the compensatory role of informal learning channels such as intergenerational support and community training programs. Scores among those with high school diplomas or higher education levels have stabilized, indicating that secondary education has reached the threshold for digital competence, with higher education yielding no additional marginal gains. Cultural welfare exhibits significant stratification by educational attainment (*F* = 2.711, *p* = 0.022): the group with a college education or higher had significantly higher scores than other groups, highlighting the distinct advantage of higher-educated individuals in accessing institutional cultural resources. Meanwhile, the elementary school group faces a significant deficit in cultural welfare access, exposing how deficiencies in basic education limit their ability to identify and utilize such resources. Well-being increases with educational attainment (*F* = 2.829, *p* = 0.017), with those holding associate degrees or higher reporting 16.5% greater well-being than high school graduates. This reflects the combined benefits of economic security, social recognition, and cultural privilege.

**Table 7:** Comparison of scale scores across educational levels (mean ± standard deviation).

Educational attainment	Sample size	Digital Disability Scale	Cultural welfare deprivation scale	Subjective Well-Being Scale
No formal education	14	85.57 ± 12.35	53.86 ± 9.61	61.36 ± 7.79
Literate but no formal education	4	94.75 ± 7.14	61.50 ± 7.51	56.75 ± 2.50
Elementary school	58	85.95 ± 12.85	51.50 ± 9.63	63.07 ± 10.60
Junior high school	62	95.31 ± 13.34	54.19 ± 9.64	60.11 ± 10.25
High school/vocational school	36	93.64 ± 16.17	55.64 ± 12.08	63.19 ± 11.71
College or higher	8	94.88 ± 8.68	63.25 ± 6.63	73.63 ± 8.48
*F*		3.798	2.711	2.829
*p*		0.003**	0.022*	0.017*

As shown in Table 8:

**Table 8:** Comparison of scale scores across different residential types (mean ± standard deviation).

Residence	Sample size	Digital Disability Scale	Cultural welfare deprivation scale	Subjective Well-Being Scale
Rural areas	115	88.82 ± 14.59	52.61 ± 10.67	61.54 ± 9.93
Urban communities	60	95.77 ± 11.73	56.93 ± 9.22	64.23 ± 11.92
Migrant populations (accompanying children or working)	7	91.57 ± 14.41	55.71 ± 7.93	57.71 ± 8.06
*F*		5.069	3.681	1.959
*p*		0.007**	0.027*	0.144

Digital disability (reverse-scored) exhibits significant residential disparities (*F* = 5.069, *p* = 0.007), with urban communities scoring highest, rural areas lowest, and migrant populations in between. The urban–rural gap exposed spatial imbalances in infrastructure and educational resources, while the “semi-integration dilemma” among migrant groups was particularly pronounced. This may be linked to insufficient digital skill acquisition due to language barriers or social isolation. Notably, severe differentiation existed within the rural group (standard deviation ± 14.59), indicating the coexistence of a minority of digitally proficient individuals alongside widespread digital illiteracy, reflecting localized fractures in digital competence.

Access to cultural welfare exhibits a tiered distribution pattern among the three groups: urban communities > migrant populations > rural areas (*F* = 3.681, *p* = 0.027). Urban areas leverage resources such as museums and senior universities to establish advantages, while rural regions remain disadvantaged due to insufficient cultural facilities. Although migrant groups formally participate in urban activities, they struggle to achieve cultural fulfillment due to a lack of cultural identification—for instance, unfamiliarity with local dialects and geographical settings.

There was no significant difference in well-being levels across residential areas (*F* = 1.959, *p* = 0.144), but latent differentiation was evident. Urban communities had the highest average scores, while migrant populations scored the lowest. Rural residents exhibited a “low resources-high well-being” phenomenon, likely due to traditional social relationships like kinship, mutual aid, and neighborhood bonds buffering negative emotions. Migrant groups, however, faced dual psychological costs—disconnected from their rural cultural roots yet unintegrated into urban cultural systems—resulting in well-being 6.2% lower than that of rural residents.

### 5.3 Linear regression analysis

As shown in Table 9, subjective well-being exhibits significant positive correlations with both digital disability (reverse-scored) (*r* = 0.169, *p* < 0.05) and cultural welfare deprivation (reverse-scored) (*r* = 0.254, *p* < 0.01). However, the strength of the association with cultural welfare deprivation is 1.5 times that of digital disability, highlighting the greater well-being benefits of cultural participation. Thus, research hypothesis 1, 2 and 3 are supported.

**Table 9:** Pearson correlation analysis results between subjective well-being and various variables.

Variable	Subjective well-being
Digital disability	0.169*
Cultural welfare deprivation	0.254**

*p < 0.05; **p < 0.01.

Table 10 indicates that only cultural welfare exhibits a significant positive relationship with subjective well-being, while none of the control variables demonstrated significant predictive power. After controlling for age, gender, and educational attainment, cultural welfare access still significantly predicted well-being (*β* = 0.221, *p* = 0.032), while the effect of digital adaptation disappeared (*β* = 0.023, *p* = 0.846). This indicates that access to cultural welfare is a more stable and direct influencing factor. Although digital disability showed a significant correlation with well-being in the correlation analysis, its effect was insignificant in the regression model. This suggests that digital disability may indirectly influence well-being by empowering cultural participation rather than directly generating psychological utility. The results of the multiple regression analysis indicate that when digital literacy and cultural welfare are simultaneously included in the model, only cultural welfare remains significant. Therefore, Hypothesis 3 is partially supported. This finding suggests that the effect of digital literacy may be mediated through its interaction with cultural welfare, leading to the subsequent examination of Hypothesis 4.

**Table 10:** Linear regression analysis results for subjective well-being (*n* = 182).

Variable	Non-standardized coefficient		Standardized coefficient	*t*	*p*	Collinearity diagnosis	
	*B*	Standard error	Beta			VIF	Tolerance
Constant	1.313	0.36	—	3.645	0.000**	—	—
Cultural welfare deprivation	0.174	0.081	0.221	2.157	0.032*	2.017	0.496
Digital disability	0.019	0.096	0.023	0.195	0.846	2.642	0.378
Age	0.03	0.026	0.112	1.139	0.256	1.836	0.545
Gender	0.062	0.064	0.073	0.977	0.33	1.077	0.929
Educational attainment	0.051	0.029	0.14	1.754	0.081	1.217	0.822
Housing status	−0.013	0.046	−0.022	−0.285	0.776	1.095	0.913
Source of income	0.045	0.033	0.118	1.376	0.171	1.411	0.709
Health status	0.153	0.107	0.113	1.433	0.154	1.193	0.838
Parental relationship	−0.042	0.049	−0.064	−0.851	0.396	1.079	0.927
*R* ^2^	0.102						
Adjusted *R*^2^	0.055						
*F*	*F* (9,172) = 2.159, *p* = 0.027						
*D*-*W* value	1.017						

Dependent variable = Subjective well-being. *p < 0.05, **p < 0.01.

### 5.4 Analysis of modulating effects

To examine the moderating effect of cultural welfare on the relationship between digital disability and subjective well-being, this study employed hierarchical regression analysis. After controlling for demographic variables, the independent variable (digital disability) and moderator variable (cultural welfare) were sequentially introduced, with their interaction term ultimately included in the model. Table 11 indicates that the interaction term’s regression coefficient is significant (*β* = −0.176, *p* < 0.05), and the model’s explanatory power (Δ*R*^2^) significantly improves (Δ*F* = 5.328, *p* = 0.022). This confirms the moderating effect of cultural deprivation. Research hypothesis 4 holds.

**Table 11:** Stratified regression analysis results for the moderating effect of cultural welfare deprivation (*n* = 182).

Variable	Model1					Model2					Model3				
	*B*	Standard error	*t*	*p*	*β*	*B*	Standard error	*t*	*p*	*β*	*B*	Standard error	*t*	*p*	*β*
Constant	1.695	0.273	6.207	0.000**	—	1.813	0.274	6.609	0.000**	—	1.844	0.271	6.799	0.000**	—
Digital disability level	0.146	0.071	2.047	0.042*	0.179	−0.008	0.097	−0.08	0.937	−0.009	−0.03	0.096	−0.317	0.751	−0.037
Mean cultural welfare deprivation						0.187	0.081	2.327	0.021*	0.238	0.183	0.08	2.304	0.022*	0.233
Mean digital competence scale score × mean cultural welfare											−0.252	0.109	−2.308	0.022*	−0.176
*R* ^2^	0.09					0.118					0.145				
Adjusted *R*^2^	0.043					0.067					0.09				
*F*-value	*F* (9,172) = 1.898, *p* = 0.055					*F* (10,171) = 2.294, *p* = 0.015					*F* (11,170) = 2.622, *p* = 0.004				
Δ*R*^2^	0.09					0.028					0.027				
Δ*F*-value	*F* (9,172) = 1.898, *p* = 0.055					*F* (1,171) = 5.413, *p* = 0.021					*F* (1,170) = 5.328, *p* = 0.022				

Dependent variable = Subjective well-being. *p < 0.05, **p < 0.01.

**Figure 1:**
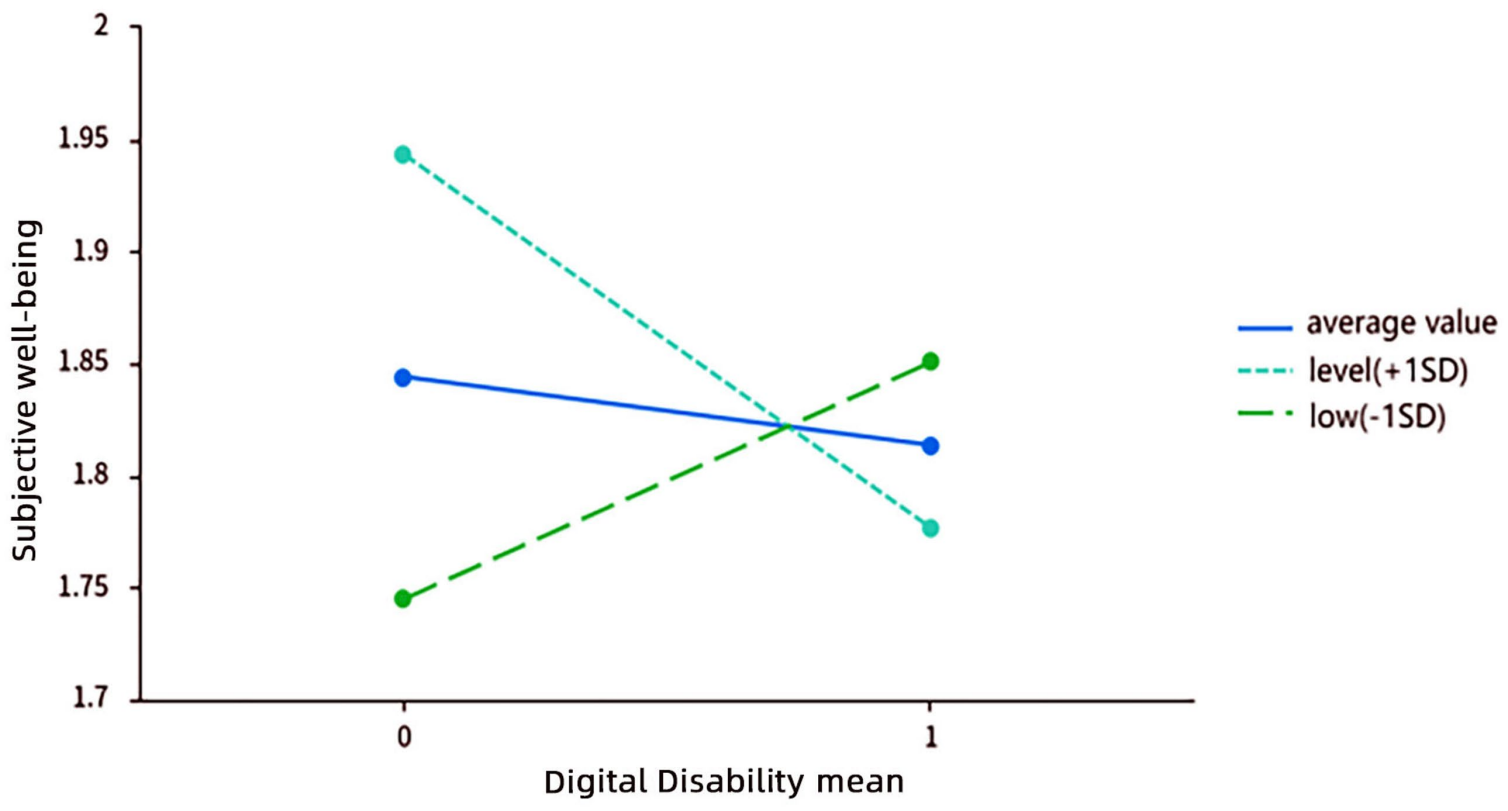
Simple slope plot of the moderating effect of cultural welfare deprivation.

To delve deeper into the specific mechanisms underlying this moderating effect of cultural deprivation, we conducted a simple slope analysis, as shown in Figure 1 and Table 12. It is important to note that, given the current sample size, the simple-slopes for both high and low levels of cultural deprivation were not statistically significant (high level: *B* = 0.106, *p* = 0.327; low level: *B* = −0.167, *p* = 0.159). Although these coefficients lack statistical significance, their trends align closely with theoretical expectations, providing preliminary support and direction for further exploration of cultural deprivation as a boundary condition. Therefore, the following interpretation of the moderation pattern is exploratory, based on the observed coefficient trends.

**Table 12:** Results of simple slope analysis for the moderating effect of cultural welfare deprivation.

Adjust variable levels	Regression coefficient	Standard error	*t*	*p*	95% CI	
Mean	−0.03	0.096	−0.317	0.751	−0.219	0.158
High level (1SD)	−0.167	0.118	−1.415	0.159	−0.397	0.064
Low level (−1SD)	0.106	0.107	0.984	0.327	−0.105	0.316

In a context of high cultural deprivation (low cultural welfare) (*M* − 1SD), digital disability (digital competence) showed a trend of positive prediction on well-being (*B* = 0.106). This suggests that when older adults face difficulties accessing offline cultural resources, enhancing digital competence could become a potential alternative pathway to fulfill their social, recreational, and informational needs. Under these conditions, digital technology may serve a compensatory function, with relatively high marginal utility, potentially mitigating the negative impacts of cultural resource scarcity and thereby positively promoting well-being.

In low cultural deprivation (high cultural welfare) scenarios (*M* + 1SD), the impact of digital disability (digital competence) on well-being showed a negative trend (*B* = −0.167). This may occur because when older adults’ cultural needs are already sufficiently met through offline channels, the added value of digital technology becomes relatively limited. At this point, excessive or inefficient digital engagement could produce the following effects: First, resource displacement, reducing time available for high-quality offline activities; second, technological pressure, where learning complex functions may lead to frustration; third, potential risks, such as facing online fraud or privacy breaches. These factors might collectively diminish the marginal utility of digital technology in this context, even showing a potential inhibitory trend.

A key finding of this study is that the level of cultural deprivation serves as a critical boundary condition for the relationship between digital competence and well-being. The confirmation of this moderating effect indicates that the impact of digital technology on the well-being of older adults is not linearly consistent but depends on their cultural resource environment. While the specific simple slopes were not significant the overall pattern-where digital competence may be more beneficial in high-deprivation contexts and less so in low-deprivation contexts provides a valuable theoretical framework and clear direction for future research. The exploratory findings of a “compensatory effect in culturally deprived contexts” and an “inhibitory trend in culturally enriched contexts” warrant further investigation with larger samples.

## 6 Countermeasures and recommendations

Based on the “technology-culture-psychology” synergistic framework and the aforementioned conclusions, the following tiered and targeted policy recommendations are proposed:

First, consolidate the digital foundation and implement a differentiated strategy aimed at reducing the digital disability rate.

Given the fundamental impact of digital disability on the construction of a digital society and the significant internal disparities within the group, policies should avoid a one-size-fits-all approach. At the national level: Incorporate reducing the digital disability rate among older adults into the strategies for aging cause development and public cultural services to provide top-level design for policy formulation. For individuals with severe disabilities, provide one-on-one, personalized, hands-on guidance to prioritize addressing basic survival needs such as scanning QR codes, making payments, and registering for appointments. For those with moderate or mild disabilities, offer advanced courses like smartphone photography and short video production to meet their social and self-actualization needs. Social and Family Level: Encourage universities and enterprises to establish digital volunteer teams for seniors, providing targeted services in communities; advocate for family members to assume the responsibility of “intergenerational tech mentoring” to alleviate technological anxiety among the older adults.

Second, enhance cultural offerings and establish an integrated online and offline cultural welfare system.

Cultural welfare offers a more direct pathway to enhancing well-being and can moderate the effects of digital technology. Offline initiatives: In urban areas, promote age-friendly renovations of public cultural venues (museums, libraries, art galleries), maintain physical service counters and printed guides, and organize dedicated events for senior citizens. In rural areas, the problem of “perfunctory implementation” activities should be focused on and tackled. By leveraging village-level cultural activity stations, local performing arts groups should be cultivated to organize sustainable cultural events tailored to seniors’ interests (such as touring opera performances and handicraft workshops). Online Initiatives: Promote “age-friendly content” by encouraging cultural institutions and tech companies to collaborate on developing apps or mini-programs that aggregate senior-friendly content (such as health education, traditional opera and folk arts, nostalgic films and TV shows). Implement comprehensive age-friendly adaptations (large fonts, simplified operations, ad-free interfaces, dialect voice support) to ensure digital cultural resources are truly “accessible, intuitive, and empowering.”

Third, promote collaborative empowerment and formulate targeted intervention policies based on community cultural contexts.

Policy formulation should first assess the level of cultural welfare in communities and implement a “one community, one policy” approach. For communities with high cultural deprivation (such as resource-scarce rural areas and old residential quarters), a “digital inclusion priority” strategy should be implemented. Government resources should be prioritized for these areas, with the policy objective of addressing cultural resource gaps. The initiative focuses on providing subsidies for basic smart devices, free community network coverage, and digital skills training to address fundamental living needs, aiming to maximize well-being benefits with limited investment. For communities with low cultural welfare deprivation (such as urban communities with well-developed cultural facilities): Implement a “deep integration of digital and cultural elements” model. The policy focus should shift from “teaching how to use mobile phones” to “using mobile phones to enrich and enhance existing cultural life.” For example, organizing offline event registrations through WeChat groups, showcasing senior university learning achievements via short video platforms, and developing smart guide systems for cultural venues to enhance the visitor experience. Let digital technology serve to deepen offline cultural experiences, avoiding its transformation into an isolated technological burden and source of stress.

## 7 Limitations and outlook

Despite the findings of this study, several limitations remain, which also point to areas for improvement in future in-depth research.

Limitations of Sample Representativeness: The sample coverage is geographically limited, primarily drawn from ordinary communities and rural areas, resulting in a lack of broad regional representativeness; The sample types are incomplete, failing to encompass older adults residing in high-end urban communities, institutionalized care settings, or emerging care models (such as travel-based retirement living). Due to limited research time, the final effective sample size obtained (*n* = 182) was relatively small. Future research should broaden the sampling scope to enhance the generalizability of conclusions.

Inadequate control of confounding variables: This study suffers from insufficient control variables in its exploration process. Key variables such as “economic income” and “chronic diseases” were not controlled for in this study, and these factors may simultaneously influence digital technology access, cultural participation capacity, and subjective well-being. Future research should incorporate these variables into models to more clearly identify causal relationships among variables.

Further exploration of mechanisms is required: While this study confirms the moderating effect, the mediating pathways through which digital disability influences well-being—such as social support and self-efficacy—have not been fully explored. Future research may employ structural equation modeling to further analyze these mediating mechanisms.

Future research can build upon this foundation to further explore differentiated pathways for digital inclusion among older adults across different regions and care models, thereby providing richer theoretical underpinnings and practical guidance for constructing an inclusive aging society.

## 8 Research conclusions

This study aims to systematically examine the impact of digital disability on subjective well-being among older adults and its underlying mechanisms, with a focus on testing the moderating effect of cultural deprivation. The empirical analysis supports the core hypotheses: digital disability is significantly correlated with subjective well-being, and the level of cultural deprivation has a significant moderating effect on this relationship. Specifically, digital disability shows a differential distribution within the older population based on age, educational attainment, and place of residence. Linear regression indicates that cultural deprivation (inverse) is a more stable and direct predictor of subjective well-being than digital disability (inverse). Further analysis of the moderating effect reveals that under high cultural deprivation (low cultural resources), the reduction in digital disability exerts a stronger positive impact on well-being, where digital technology plays a crucial “compensatory effect.” Conversely, under low cultural deprivation (high cultural resources), this positive impact weakens or even shows a “redundant effect.” This indicates that discussing the effects of digital empowerment without considering the cultural welfare context presents an incomplete picture. The Digital Disability Scale and Cultural Deprivation Scale developed in this study demonstrate good reliability and validity, providing reliable localized measurement tools for related research.

In summary, this study not only reveals the mechanisms and boundary conditions through which digital competence and cultural welfare affect the well-being of older adults but also provides theoretical foundations and practical implications for precisely enhancing the well-being of this population in the digital era.

## Data Availability

The original contributions presented in the study are included in the article/Supplementary material, further inquiries can be directed to the corresponding author.

## References

[1] Li W. Analysis on the influence factors and structural equation models of subjective well-being of community elderly-based on Chaoyang district of Changchun city. Changchun: Jilin University (2017).

[2] Yan Y. The influence of internet use on life satisfaction of the elderly. Changchun: Jilin University (2022).

[3] Wang Y. Research on new media use and happiness enhancement of the elderly. Qingdao: Qingdao University (2023).

[4] Xu Z Hu A Huang L. A review of literature on the elderly’s internet usage behavior. Libr Inf Serv. (2017) 61:140–8. doi: 10.13266/j.issn.0252-3116.2017.20.015

[5] Li Y. Research on cultural welfare development of senior citizens in Shalang bai people communities of Kunming under urbanization. Kunming: Yunnan University (2016).

[6] Wei Y. Research on the construction of cultural welfare in minority areas. Guiyang: Guizhou University of Finance and Economics (2015).

[7] Shan N Zhang X. Research on the digital disability and measurement indicators of the elderly in the digital divide. Sci Res Ageing. (2023) 11:50–62. doi: 10.3969/j.issn.2095-5898.2023.08.006

[8] Shan D. Where the road leads: an exploration of the impact of digital disability on the employment relationship of overage migrant workers. Lanzhou Acad J. (2023):149–60. doi: 10.3969/j.issn.1005-3492.2023.09.011

[9] Wang B. Characteristics, causes and governance of digital disempowerment among the aged: a perspective on digital dividends. J Yunnan Minzu Univ. (2024) 41:60–7. doi: 10.13727/j.cnki.53-1191/c.20240305.006

[10] Wang B. Prevalence and determinants of digital disability of urban and rural older adults in China: based on the multidimensional explanatory framework of digital inequality. J Zhejiang Gongshang Univ. (2024) 5:156–68. doi: 10.14134/j.cnki.cn33-1337/c.2024.05.014

[11] Ariyanti O Sampaio D Bailey A. Older adults’ experiences with app-based ride-hailing in Indonesia: at the intersection of health, gender, digital literacy, and affordability. J Transp Health. (2025) 44:102134. doi: 10.1016/j.jth.2025.102134

[12] Srisaknok T Ploylearmsang C Wongkongdech A Chanthamath S Wongkongdech R. Health literacy and self-medication among older adults in rural Thailand: understanding inequities in a digital era. Glob Health Action. (2025) 18:2572008. doi: 10.1080/16549716.2025.2572008, 41090235 PMC12529737

[13] Pituk P Chutipattana N Laor P Sukdee T Kittikun J Jitwiratnukool W . Digital media victimization among older adults in upper-southern Thailand. Informatics. (2025) 12:24. doi: 10.3390/informatics12010024

[14] Liu W. Digital competence problems and coping strategies of rural elderly. Rural Area Agri Farm. (2023) 24:37–9. doi: 10.3969/j.issn.1003-6261.2023.24.013

[15] Yang D Chen X. The impact and mechanism of digital disempowerment on elderly’s well-being. J Gansu Admin Inst. (2024):80–9. doi: 10.3969/j.issn.1009-4997.2024.06.008

[16] Zhang J. The research on influencing factors of elderly subjective well-being in China. Jinan: Shandong University (2014).

[17] Mechanic D Bradburn NM. The structure of psychological well-being. Am Sociol Rev. (1970) 35:948–9. doi: 10.2307/2093340

[18] Diener E. Subjective well-being. Psychol Bull. (1984) 95:542–75. doi: 10.1037/0033-2909.95.3.542 6399758

[19] Wang C. Study on cultural elderly care and subjective well-being of the elderly. Jinan: Shandong University (2023).

[20] Cai Y Jing Z Yue Y. Effects of intergenerational support from children on depression in rural empty nesters. Chinese J Clinic Res. (2024) 37:1905–9. doi: 10.13429/j.cnki.cjcr.2024.12.018

[21] Malone C Wachholtz A. The relationship of anxiety and depression to subjective well-being in a mainland Chinese sample. J Relig Health. (2017) 57:266–78. doi: 10.1007/s10943-017-0447-4, 28702737 PMC5764815

[22] Tang L Mila Y Hu Y Zhang H. Relationship of optimistic personality with depression and subjective well-being in older adults. Chin J Gerontol. (2022) 42:1195–7. doi: 10.3969/j.issn.1005-9202.2022.05.049

[23] Blanchflower DG. Is happiness U-shaped everywhere? Age and subjective well-being in 145 countries. J Popul Econ. (2020) 34:575–624. doi: 10.1007/s00148-020-00797-z, 32929308 PMC7480662

[24] Latten JJ. Life-course and satisfaction, equal for every-one? Soc Indic Res. (1989) 21:599–610. doi: 10.1007/bf02217995

[25] Zheng F Lu Y. Income satisfaction: turning point estimation and policy guidance an empirical analysis based on Guangdong province from 2007 to 2011. South China J Econ. (2013):78–89. doi: 10.19592/j.cnki.scje.2013.08.008

[26] Sum S Mathews RM Pourghasem M Hughes I. Internet use as a predictor of sense of community in older people. Cyberpsychol Behav. (2009) 12:235–9. doi: 10.1089/cpb.2008.0150, 19250013

[27] Shapira N Barak A Gal I. Promoting older adults’ well-being through internet training and use. Aging Ment Health. (2007) 11:477–84. doi: 10.1080/13607860601086546, 17882585

[28] Tabet ER. Activity participation and older adults’ well-being. Educ Citizen Soc Justice. (2016) 2:22–8.

[29] Sabatini F Sarracino F. Online networks and subjective well-being. Kyklos. (2017) 70:456–80. doi: 10.1111/kykl.12145

[30] Yang H. Effects of internet use on subjective well-being among older adults: a chain mediating effect of loneliness and attitudes towards aging. Xinjiang: Shihezi University (2023).

[31] Liu X. The mediation of social support and self-esteem in the subjective well-being of elderly people is impacted by internet usage. Xi’an: Shanxi Normal University (2023).

[32] Deng J. Research on the happiness of rural elderly based on digital literacy: a case study of H town in Pengshan district. Xianyang: Northwest A&F University (2023).

[33] Ménard A Maharaj A Harb S Fraser S O’Sullivan T. Representations of older adults’ digital literacy in Canadian news media: critical discourse analysis using unified theory of acceptance and use of technology 2. JMIR Aging. (2025) 8:e69373. doi: 10.2196/69373, 40882616 PMC12396828

[34] Toepoel V. Ageing, leisure, and social connectedness: how could leisure help reduce social isolation of older people? Soc Indic Res. (2012) 113:355–72. doi: 10.1007/s11205-012-0097-6, 23874058 PMC3696179

[35] Kim EJ Park SM Kang HW. Changes in leisure activities of the elderly due to the COVID-19 in Korea. Front Public Health. (2022) 10:966989. doi: 10.3389/fpubh.2022.966989, 36033753 PMC9412193

[36] Rezaeipandari H Ravaei J Bahrevar V Mirrezaei S Morowatisharifabad MA. Social participation and loneliness among older adults in Yazd, Iran. Health Soc Care Community. (2020) 28:2076–85. doi: 10.1111/hsc.13018, 32483925

[37] Liu H. The construction of social and cultural welfare in the era of mass communication. Youth J. (2015) 30:44–6. doi: 10.15997/j.cnki.qnjz.2015.30.026

[38] Hou Z Fu M Sun Q. The rural culture of welfare capital and the culture of welfare governance. J South China Agri Univ. (2013) 12:107–13.

[39] Zhang Q. The study of farmers’ cultural welfare. Shijiazhuang: Hebei University of Economics and Trade (2015).

[40] Chen S. The great practice of the construction of public digital cultural resources. Libr J. (2015) 34:4–12. doi: 10.13663/j.cnki.lj.2015.11.001

[41] Zhao J Li S. Cultural welfare at grassroots level: peasants’ weak participation and governance prospect. Libr Develop. (2019):79–89. doi: 10.19764/j.cnki.tsgjs.20182655

[42] Zheng F Ruan R. Farmers’ recreation well-being and recreation demand: based on fieldwork from song county in Henan province. J Tianjin Univ Commerce. (2010) 30:3–7. doi: 10.15963/j.cnki.cn12-1401/f.2010.03.009

[43] Shen W. Research on the multi-subject interactive relationship of the cultural welfare supply in urban community: an example of H community in Chongqing. Chongqing: Chongqing Technology and Business University (2021).

[44] Pan X. Research on cultural welfare development for the elderly in urban communities: a case study of sunshine community in Guiyang city. Guiyang: Guizhou University of Finance and Economics (2017).

[45] Baek EM Choi B Kang SH. Exploring the relationship between digital competency and life satisfaction among the elderly. Digital Health. (2025) 11:20552076251358756. doi: 10.1177/20552076251358756, 40893171 PMC12394858

[46] He T Huang C Li M Zhou Y Li S. Social participation of the elderly in China: the roles of conventional media, digital access and social media engagement. Telematics Inform. (2020) 48:101347. doi: 10.1016/j.tele.2020.101347

[47] Zhang M Liu C Tang J Liu X. Influence mechanism of digital reading on digital integration of the elderly. Digit Libr Forum. (2024) 20:80–90. doi: 10.3772/j.issn.1673-2286.2024.10.009

[48] Deng X. Can social media use improve the elderly subjective well-being: a survey based on the active aging background. Doc Info Know. (2021) 38:77–94. doi: 10.13366/j.dik.2021.05.077

[49] Hua Y Chen Y Wang M. Study on user demands for digital construction of public cultural services in rural areas of eastern China: taking Jiangsu province as an example. J Nation Libr China. (2023) 32:61–71. doi: 10.13666/j.cnki.jnlc.2023.0606

[50] Huang L. From digital divide to digital inclusion: study on the inclusion strategy of public cultural digital services for the elderly people. Libr Dev. (2024):120–7. doi: 10.19764/j.cnki.tsgjs.20240938

[51] Xin Y Chen Y. Digital integration of “silver-haired surfers” from the perspective of short video. Jiangsu Soc Sci. (2024) 5:214–22. doi: 10.13858/j.cnki.cn32-1312/c.20240918.007

[52] Wang Y. A study on the influence of social media practice of elderly internet celebrities on subjective well-being. Hangzhou: Zhejiang University of Media and Communications (2024).

[53] Cao Y Zhang J. Research on the logical mechanism of high-quality development of rural public culture with digital empowerment. New Cent Libr. (2024) 6:46–53. doi: 10.16810/j.cnki.1672-514X.2024.06.007

[54] Ohiri SC Ihebom D Nnennaya C. Psychometric properties of a test: an overview. Int J Res Publ Rev. (2024) 5:2217–24. doi: 10.55248/gengpi.5.0224.0539

[55] Kozma A Stones MJ. The measurement of happiness: development of the Memorial University of Newfoundland scale of happiness (MUNSH). J Gerontol. (1980) 35:906–12. doi: 10.1093/geronj/35.6.906, 7440930

[56] Zhu J. Study on the influence of social support on subjective well-being of the elderly residents in Chinese urban. Hefei: Hefei University of Technology (2020).

[57] Ren W. The impact of intergenerational support on the subjective well-being of the elderly: the chain mediating effect of social isolation and aging. Huaibei: Huaibei Normal University (2023).

[58] Che J. Research on the supply of cultural endowment services for the elderly in rural communities from the perspective of active aging: a case study of Tongtan town, Luzhou city. Kunming: Yunnan University of Finance and Economics (2023).

[59] Vega OA Arroyave-Zambrano PM Ocampo-Arias J Sánchez-Velásquez SP. Inclusión digital como opción aportante al envejecimiento activo. E-Ciencias Info. (2020) 10:6–8. doi: 10.15517/eci.v10i2.39522

