# Supplementary material for: The impact of digital disability on the well-being of older adults: the moderating role of cultural deprivation
Source: Front Public Health. 2026 Feb 19;14:1713695. doi: 10.3389/fpubh.2026.1713695 (PMC12961692; doi:10.3389/fpubh.2026.1713695)
Supplement: Supplementary file 1 [file Data_Sheet_1.zip › New Data Sheet/Appendix A: Scale Development.docx]

Appendix: Scale Development

# Development of a Scale for Measuring the Degree of Digital Disability in Older Adults

## Research Objectives

Given the issue of the "digital divide" faced by the elderly population, exploring the current state of digital disability among older adults and its relationship with well-being is of great significance. However, previous studies have found that existing scales focusing on the degree of digital disability in older adults, both domestically and internationally, still need further development. To gain a clearer understanding of the degree of digital disability in older adults, specifically regarding smartphone usage, Study 1 aims to design and develop a scale to assess the degree of digital disability in older adults. It is expected to provide an effective measurement tool for research in related fields and further promote the development of studies on the relationship between digital disability and the well-being of the elderly population in the context of the digital divide.

## Development Process

### Literature Review and Theoretical Framework

Although there are currently no detailed scales for measuring digital disability domestically or internationally, relevant scholars have made significant breakthroughs in indicators for measuring digital disability. South Korean scholar Woochun Jun constructed primary indicators of digital disability for specific groups based on individuals' engagement with computer environments. Scholar Zhang and others innovatively developed a "digital disability" indicator, selecting "frequency of online shopping" as a manifestation of digital competence among elderly migrant workers in the digital age, though this indicator is relatively singular. Scholars such as Shan categorized the degree of digital disability into three levels within the digital divide's "access gap, usage gap, and knowledge gap": lacking digital competence, partially lacking digital competence, and relatively lacking digital competence. These have all provided the most basic indicator references for the development of this research. By reviewing domestic and international studies on digital inclusion, the Technology Acceptance Model (TAM), and digital feedback, and combining China's "aging + digitalization" social characteristics, preliminary indicators of digital disability are proposed (see Table 1).

Table 1: Indicators of Digital Disability in Older Adults

| Target Layer | Dimension Layer | Indicator Layer |
| --- | --- | --- |
| Indicators of Digital Disability in Older Adults (A) | Digital Access(B1) | Device Accessibility（C1） |
|  |  | Network Coverage（C2） |
|  | Digital Skills(B2) | Basic operational skills（C3） |
|  |  | Communication skills（C4） |
|  |  | Information Security and Privacy Protection Skills（C5） |
|  |  | Problem-solving skills（C6） |
|  |  | IInformation Interaction Skills（C7） |
|  | Digital Usage（B3） | Frequency of Use（C8） |
|  |  | Versatility in usage scenarios（C9） |

### AHP Expert Group Decision-Making and Indicator Weight Setting

Based on the indicators formed in the previous step, to ensure the scientificity and validity of the indicator construction, an expert group consisting of 2 nursing home managers and 3 community digital service experts was invited. The Analytic Hierarchy Process (AHP) was used to conduct pairwise comparisons of the importance of indicators in each dimension, construct judgment matrices, and calculate weights. The consistency test results showed that the CR values of all judgment matrices were less than 0.1 (see Table 2 and Table 3), indicating a high degree of consistency among expert opinions.

Table 2: Consistency Test Results of AHP Judgment Matrix for Intermediate Layer Elements

| Intermediate Layer Elements | Eigenvector | Weight | Ranking | Maximum Eigenvalue | CI | RI | CR | Consistency Test Result |
| --- | --- | --- | --- | --- | --- | --- | --- | --- |
| Digital Access | 0.6594 | 0.6594 | 1 | 3.0869 | 0.0435 | 0.58 | 0.0750 | Passed |
| Digital Skills | 0.2105 | 0.2105 | 2 | 5.2967 | 0.0742 | 1.12 | 0.0663 | Passed |
| Digital Usage | 0.1301 | 0.1301 | 3 | 2.0000 | 0.0000 | 0.00 | 0.0000 | Passed |

Table 3: Consistency Test Results of AHP Judgment Matrix for Scheme Layer Elements

| Scheme Layer Elements | Eigenvector | Weight | Ranking | Maximum Eigenvalue | CI | RI | CR | Consistency Test Result |
| --- | --- | --- | --- | --- | --- | --- | --- | --- |
| Device Accessibility | 0.3956 | 0.3956 | 1 | 3.0539 | 0.0269 | 0.58 | 0.0464 | Passed |
| Network Coverage | 0.2637 | 0.2637 | 2 | 2.0000 | 0.0000 | 0.00 | 0.0000 | Passed |
| Basic operational skills (physical contact and simple interaction) | 0.0755 | 0.0755 | 3 | 5.3966 | 0.0991 | 1.12 | 0.0885 | Passed |
| Communication skills | 0.0729 | 0.0729 | 4 | 2.0000 | 0.0000 | 0.00 | 0.0000 | Passed |
| Information Security and Privacy Protection Skills | 0.0573 | 0.0573 | 5 | 2.0000 | 0.0000 | 0.00 | 0.0000 | Passed |
| Problem-solving skills | 0.0518 | 0.0518 | 6 | 5.4352 | 0.1088 | 1.12 | 0.0972 | Passed |
| Information Interaction Skills | 0.0404 | 0.0404 | 7 | 5.2426 | 0.0607 | 1.12 | 0.0542 | Passed |
| Frequency of Use | 0.0226 | 0.0226 | 8 | 5.4009 | 0.1002 | 1.12 | 0.0895 | Passed |
| Versatility in usage scenarios | 0.0202 | 0.0202 | 9 | 5.2967 | 0.0742 | 1.12 | 0.0663 | Passed |

### Analysis of Preliminary Questionnaire Results

After completing the development of the preliminary questionnaire, members of our research team conducted a pre-survey in 5 nursing homes, 3 communities, 2 parks, and 1 administrative village in Jinshui District and Huiji District of Zhengzhou City. A total of 200 questionnaires were distributed, and 182 valid questionnaires were recovered, which met the requirements for initial analysis. Reliability analysis and construct validity analysis were performed on these 182 questionnaires.

#### Exploratory Factor Analysis

To summarize the complex and diverse scale items into a few core factors while eliminating non-standard items, thereby improving the overall quality of the scale, further exploratory factor analysis will be conducted. However, before conducting exploratory factor analysis, it is necessary to ensure that the data meet the established statistical standards. Therefore, the significance test of Bartlett's test of sphericity is a prerequisite for factor analysis. In the preliminary scale of this study, Bartlett's test of sphericity was significant (p < 0.001), and the KMO values were all greater than 0.7, which fully indicates that the data are suitable for exploratory factor analysis. For specific data, see Table 4.

Table 4 KMO and Bartlett's Test of Sphericity

| Name | KMO | Bartlett's Test of Sphericity | | |
| --- | --- | --- | --- | --- |
| Digital Disability | 0.933 | χ2 | df | p |
|  |  | 5898.379 | 351 | 0.000 |
| Digital Access | 0.735 | χ2 | df | p |
|  |  | 666.048 | 6 | <0.001 |
| Digital Skill | 0.945 | χ2 | df | p |
|  |  | 3676.089 | 153 | 0.000 |
| Digital Usage | 0.774 | χ2 | df | p |
|  |  | 762.438 | 10 | <0.001 |

The principal component analysis method was adopted. In accordance with the Kaiser criterion, five common factors with eigenvalues greater than 1 were extracted. These factors serve as 2 measurement dimensions for digital access, 5 measurement dimensions for digital skill, and 2 measurement dimensions for digital usage within the degree of digital disability in older adults. Orthogonal rotation was performed using the maximum variance method to derive clearer relationships between variables. A total of 24 items with absolute factor loadings greater than 0.5 were retained, and the factor loading matrix was obtained (see Table 5, Table 6, and Table 7), which serve as the measurement indicators for older adults' community satisfaction.

Table 5 Factor Loading Matrix for Digital Access

| Item | F1 | F2 |
| --- | --- | --- |
| 16-1 | 0.901 |  |
| 16-2 | 0.871 |  |
| 15-2 |  | 0.896 |
| 15-1 |  | 0.818 |

Table 6 Factor Loading Matrix for Digital Skill

| Item | F1 | F2 | F3 | F4 | F5 |
| --- | --- | --- | --- | --- | --- |
| 21-4 | 0.806 |  |  |  |  |
| 21-3 | 0.799 |  |  |  |  |
| 21-2 | 0.787 |  |  |  |  |
| 21-5 | 0.694 |  |  |  |  |
| 21-1 | 0.600 |  |  |  |  |
| 19-3 |  | 0.823 |  |  |  |
| 19-2 |  | 0.809 |  |  |  |
| 19-1 |  | 0.686 |  |  |  |
| 19-4 |  | 0.685 |  |  |  |
| 17-1 |  |  | 0.853 |  |  |
| 17-3 |  |  | 0.838 |  |  |
| 17-2 |  |  | 0.777 |  |  |
| 18-2 |  |  |  | 0.739 |  |
| 18-3 |  |  |  | 0.687 |  |
| 18-1 |  |  |  | 0.657 |  |
| 20-2 |  |  |  |  | 0.758 |
| 20-3 |  |  |  |  | 0.694 |
| 20-1 |  |  |  |  | 0.679 |

Table 7 Factor Loading Matrix for Digital Usage

| Item | F1 | F2 |
| --- | --- | --- |
| 23-1 | 0.872 |  |
| 23-3 | 0.867 |  |
| 23-2 | 0.779 |  |
| 22-2 |  | 0.906 |
| 22-1 |  | 0.900 |

#### Item Analysis and Retention

After factor analysis, the simplified and redesigned scale structure still consists of three dimensions with 27 items: Digital Access, Digital Skill, and Digital Usage.

To ensure the psychometric quality of the scale, we conducted a rigorous Exploratory Factor Analysis (EFA) on the initially developed Digital Disability Scale for Older Adults (27 initial items). The analysis adopted the principal component extraction and varimax rotation methods, and all items were evaluated based on the following criteria: 1) Factor loading must be higher than 0.50; 2) No significant cross-loading (loading on other factors is below 0.40 with a loading difference greater than 0.20); 3) Communality is higher than 0.30; 4) The final factor structure is consistent with the theoretical model.

The analysis results showed that all 27 items performed excellently. The factor loading of each item on its preset dimension was higher than 0.65 (see Appendix Tables 5, 6, and 7 for details), no item exhibited confusing cross-loading, and the communality was good. The three extracted common factors clearly corresponded to the theoretical structures of "Digital Access", "Digital Skills", and "Digital Usage", with an ideal cumulative variance explained.

Therefore, all initial items met or even exceeded the preset retention criteria, indicating that the initial scale has excellent construct validity and item quality. Thus, all items were retained to form the final version of the scale.

Digital Access: It includes two secondary indicator systems: device accessibility and network coverage, with a total of 4 items. Responses to the items range from "Strongly Disagree" to "Strongly Agree," scored 1 to 5, respectively, with a total score range of 4-20.

Digital Skill: It includes five secondary indicator systems: basic operation ability, communication ability, information security and privacy protection ability, problem-solving ability, and information interaction ability, with a total of 18 items. Responses to the items range from "Strongly Disagree" to "Strongly Agree," scored 1 to 5, respectively, with a total score range of 18-90.

Digital Usage: It includes two secondary indicator systems: usage frequency and diversity of usage scenarios, with a total of 5 items. Responses to the items range from "Strongly Disagree" to "Strongly Agree," scored 1 to 5, respectively, with a total score range of 5-25.

The actual total score range of the adjusted Digital Disability Scale is 27-135, where a total score < 70 indicates severe digital disability; a total score between 70 and 100 (inclusive) indicates moderate digital disability; and a total score > 100 indicates mild digital disability.

#### Reliability Analysis

SPSS 27.0 was used to conduct reliability analysis on all questionnaire items, with no missing values. The Alpha model was adopted, and the resulting Cronbach's Alpha value was 0.975, with the standardized value also being 0.975 (see Table 8). Both coefficient values are greater than 90%, indicating that the scale has high internal consistency and strong reliability.

Table 9 Cronbach's Alpha Coefficients for the Scale of Digital Disability in Older Adults

| Cronbach’s Alpha | Cronbach's Alpha Based on Standardized Items | Number of Items |
| --- | --- | --- |
| 0.975 | 0.975 | 27 |

The reliability coefficient of this scale is 0.975, indicating that the scale has good reliability and meets the requirements of statistical analysis.

Therefore, the design of the Scale for Measuring the Degree of Digital Disability in Older Adults was completed through the initial design, initial testing, exploratory factor analysis, Item Analysis retention, and final testing of the scale. Both the construct validity and reliability tests of the scale have achieved good results, indicating that this scale can accurately measure the degree of digital disability in older adults for this study. It is also expected to become a reliable measurement tool for researching the degree of digital disability in older adults in China.

# Development of the Cultural Welfare Scale

## Research Objectives

Against the backdrop of accelerating aging and rapid digital development, the issue of cultural welfare among older adults caused by digital disability has increasingly become an obstacle affecting their dignified and happy spiritual life. The establishment of a scale for measuring cultural deprivation in older adults aims to accurately assess the status of older adults' access to welfare in the field of digital culture and to conduct an in-depth analysis of various factors affecting their digital cultural welfare. This will provide a scientific and targeted basis for improving the well-being index of older adults in their later years and optimizing relevant policies and service provision.

## Development Process

### Literature Review and Theoretical Framework

Cultural welfare is an inherent and essential connection. There is little international research on cultural deprivation. Domestically, the "Opinions on Accelerating the Construction of a Modern Public Cultural Service System" issued by the General Office of the Central Committee of the Communist Party of China and the General Office of the State Council provides a detailed classification of existing public cultural resources, which has been supplemented by the "14th Five-Year Plan for Cultural Development". Luo elaborated on the specific content of digital cultural resources. These have all provided original references for the Cultural Deprivation Scale, and preliminary indicators of cultural deprivation have been proposed (see Table 9).

Table 10: Indicators of Cultural Deprivation

| Target Layer | Dimension Layer |
| --- | --- |
| Cultural Deprivation (a) | Online Cultural Deprivation (b1) |
|  | Access to Online Cultural Information (b2) |
|  | \| Barriers to Offline Smart Services (b3) \| \| --- \| |
|  | Perception of Cultural Welfare (b4) |

### AHP Expert Group Decision-Making and Indicator Weight Setting

Based on the indicators formed in the previous step, to ensure the scientificity and validity of the indicator construction, 5 community cultural service workers were invited to form an expert group. The consistency test results using the Analytic Hierarchy Process (AHP) showed that the CR values of all judgment matrices were less than 0.1 (see Table 10), indicating a high degree of consistency among expert opinions.

Table 11: Consistency Test Results of AHP Judgment Elements

| Online Cultural Deprivation | Access to Online Cultural Information | Barriers to Offline Smart Services | Perception of Cultural Welfare | Eigenvector | Weight | Ranking |  |  |
| --- | --- | --- | --- | --- | --- | --- | --- | --- |
| 1 | 1.93318 | 2.89732 | 4.16277 | 1.83257 | 0.45814 | 1 | Maximum Eigenvalue | 4.14028 |
| 0.51728 | 1 | 2.37144 | 3 | 1.1519 | 0.28798 | 2 | CI | 0.04676 |
| 0.34515 | 0.42168 | 1 | 0.51728 | 0.46 | 0.115 | 4 | RI | 0.89 |
| 0.24022 | 0.33333 | 1.93318 | 1 | 0.55553 | 0.13888 | 3 | CR | 0.05254 |
|  |  |  |  |  |  |  | Consistency Test |  |

### Analysis of Preliminary Questionnaire Results

After completing the development of the preliminary questionnaire, members of our research team conducted a pre-survey in 5 nursing homes, 3 communities, 2 parks, and 1 administrative village in Jinshui District and Huiji District of Zhengzhou City. A total of 200 questionnaires were distributed, and 182 valid questionnaires were recovered, which met the requirements for initial analysis. Reliability analysis and construct validity analysis were performed on these 182 questionnaires.

#### Exploratory Factor Analysis

In the initial scale of Cultural Deprivation, Bartlett's test of sphericity was significant (p < 0.001), and the KMO value was greater than 0.7, which fully indicates that the data is suitable for exploratory factor analysis. For specific data, see Table 11.

Table 12 KMO and Bartlett's Test of Sphericity

| Name | KMO | Bartlett's Test of Sphericity | | |
| --- | --- | --- | --- | --- |
| Cultural Deprivation | 0.916 | χ2 | df | p |
|  |  | 2576.473 | 210 | 0.000 |

In accordance with the Kaiser criterion, five common factors with eigenvalues greater than 1 were extracted, which serve as the four measurement dimensions in Cultural Deprivation. Orthogonal rotation was performed using the maximum variance method to obtain the factor loading matrix (see Table 12).

Table 13 Factor Loading Matrix for Digital Access

| Item | F1 | F2 | F3 | F4 |
| --- | --- | --- | --- | --- |
| 26-17 | 0.816 |  |  |  |
| 26-14 | 0.779 |  |  |  |
| 26-12 | 0.755 |  |  |  |
| 26-15 | 0.734 |  |  |  |
| 26-16 | 0.732 |  |  |  |
| 26-13 | 0.671 |  |  |  |
| 24-4 |  | 0.762 |  |  |
| 24-3 |  | 0.699 |  |  |
| 24-1 |  | 0.689 |  |  |
| 24-5 |  | 0.670 |  |  |
| 24-2 |  | 0.650 |  |  |
| 25-10 |  | 0.513 |  |  |
| 25-11 |  | 0.510 |  |  |
| 25-7 |  |  | 0.726 |  |
| 25-6 |  |  | 0.685 |  |
| 25-8 |  |  | 0.674 |  |
| 25-9 |  |  | 0.637 |  |
| 27-20 |  |  |  | 0.757 |
| 27-18 |  |  |  | 0.718 |
| 27-19 |  |  |  | 0.691 |
| 27-21 |  |  |  | 0.672 |

#### Item Analysis and Retention

The factor analysis resulted in a simplified scale with four dimensions: Online Cultural Deprivation, Access to Online Cultural Information, Barriers to Offline Smart Services, and Perception of Cultural Welfare. To improve psychometric quality, we systematically refined the items. Starting from a theoretically derived 21-item pool, we conducted item analysis and Exploratory Factor Analysis (EFA), removing items with loadings below 0.50, significant cross-loadings (difference < 0.20), or conceptual misalignment with their assigned factor.

Items 25-10 and 25-11 (loadings = 0.513 and 0.510) were deleted because their loadings were only marginally acceptable, they showed cross-loading, and, most importantly, their content did not conceptually fit the "Online Cultural Deprivation" dimension on which they loaded. This decision was made to preserve the conceptual distinctiveness and construct validity of the dimensions.

The final 19-item scale, organized into four theoretically coherent dimensions, demonstrates good content validity alongside improved construct validity and reliability. The Online "Online Cultural Deprivation" includes 5 items. Responses to the items range from "Strongly Disagree" to "Strongly Agree," scored 1 to 5, respectively, with a total score range of 5-25.

The dimension "Access to Online Cultural Information" contains 4 items. Responses to the items range from "Strongly Disagree" to "Strongly Agree," scored 1 to 5, respectively, with a total score range of 4-20.

The dimension "Barriers to Offline Smart Services" comprises 6 items. Responses to the items range from "Strongly Disagree" to "Strongly Agree," scored 1 to 5, respectively, with a total score range of 6-30.

The dimension "Perception of Cultural Welfare" comprises 2 items. Responses to the items range from "Strongly Disagree" to "Strongly Agree," scored 1 to 5, respectively, with a total score range of 2-10.

The actual total score range of the adjusted Cultural Deprivation Scale is 17-85, where a total score < 45 indicates severe Cultural Deprivation; a total score between 45 and 65 (inclusive) indicates moderate Cultural Deprivation; and a total score > 65 indicates mild Cultural Deprivation.

#### Reliability Analysis

SPSS 27.0 was used to conduct reliability analysis on all questionnaire items, adopting the Alpha model. The resulting Cronbach's Alpha value was 0.934 (see Table 13). Since the coefficient value is greater than 90%, it indicates that the scale has high internal consistency and strong reliability.

Table 14 Cronbach's Alpha Coefficients for the Cultural Deprivation Scale

| Cronbach’s Alpha | Cronbach's Alpha Based on Standardized Items | Number of Items |
| --- | --- | --- |
| 0.934 | 0.934 | 19 |

The reliability coefficient of this scale is 0.934, indicating that the scale has good reliability and meets the requirements of statistical analysis.

Therefore, the design of the Cultural Deprivation Scale was completed through the initial design, initial testing, exploratory factor analysis, Item Analysis, retention, and final testing of the scale. Both the construct validity and reliability tests of the scale have achieved good results, indicating that it can provide a reliable measurement tool for studying the degree of elderly people's enjoyment of cultural welfare.

# Memorial University of Newfoundland Scale of Happiness (MUNSH)

The scale adapted by Zhu (2020) from the one developed by Kozma was adopted. The original three-level scale (A. Yes, B. Don't know, C. No) was expanded into a five-level scale (A. Yes, B. Somewhat, C. Don't know, D. A little, E. No) according to the actual situation, and it was included as an emotional factor in subjective well-being. This scale includes four sub-dimensions: Positive Affect (PA), Negative Affect (NA), Positive Experience (PE), and Negative Experience (NE). The total score is calculated as: Total score = PA + PE - NA - NE. For the convenience of statistics, 48 is added to the total score, so the score range is (0-96). A total score < 50 indicates a low-level emotional factor, 50 ≤ total score ≤ 80 indicates a medium-level emotional factor, and 80 < total score ≤ 96 indicates a high-level emotional factor. In this study, the Cronbach's α coefficient of the scale was 0.797, the Bartlett's test of sphericity was significant (p < 0.001), and KMO = 0.822, which is greater than 0.7, indicating good construct validity. See Table 14 and Table 15 for details.

Table 15 Cronbach's Alpha Coefficients for the Memorial University of Newfoundland Scale of Happiness

| Scale | Cronbach’s Alpha | Number of Items |
| --- | --- | --- |
| Memorial University of Newfoundland Scale of Happiness | 0.797 | 24 |

Table 16 KMO and Bartlett's Test of Sphericity for the Memorial University of Newfoundland Scale of Happiness

| Name | KMO | Bartlett's Test of Sphericity | | |
| --- | --- | --- | --- | --- |
| Memorial University of Newfoundland Scale of Happiness | 0.822 | χ2 | df | p |
|  |  | 2023.061 | 276 | <0.001 |
