# Supplementary material for: The impact of digital disability on the well-being of older adults: the moderating role of cultural deprivation
Source: Front Public Health. 2026 Feb 19;14:1713695. doi: 10.3389/fpubh.2026.1713695 (PMC12961692; doi:10.3389/fpubh.2026.1713695)
Supplement: Supplementary file 1 [file Data_Sheet_1.zip › New Data Sheet/Appendix Phase II Data Analysis.docx]

Supplementary Analysis Robustness Check with an Expanded Sample (N=500)

# Attachment Notes

This appendix presents the empirical analysis results of the second-phase data from the study "The Impact of Digital Disability on the Elderly's Subjective Well-being: The Moderating Role of Cultural Deprivation," serving as a supplement and validation to the analysis of the original paper based on the initial sample (n=182). The second-phase data used the same questionnaires and measurement tools as the initial data, with a total sample size of 500 after combination, aiming to enhance the robustness and generalizability of the research conclusions.

# Data Verification

## Reliability Analysis

Table 1 **Measures of Digital Disability**

| Name | Correlation of Total Item Correction  (CITC) | α Coefficient of Deleted Items | CronbachαCoefficient |
| --- | --- | --- | --- |
| 1.1Device Accessibility-I have a smartphone that can access the internet and functions normally. | 0.782 | 0.976 | 0.977 |
| I can afford the monthly phone bill and data plan. | 0.761 | 0.977 |  |
| 1.2Network Coverage-My phone's network is strong, allowing smooth browsing of news and watching videos. | 0.764 | 0.977 |  |
| My data plan and usage time fully meet my daily needs. | 0.800 | 0.976 |  |
| 2.1Basic operational skills (physical contact and simple interaction)-I can independently use my smartphone to make and receive calls. | 0.772 | 0.976 |  |
| I am proficient in sending and receiving text messages or WeChat voice messages. | 0.793 | 0.976 |  |
| I can adjust basic settings like volume and brightness. | 0.786 | 0.976 |  |
| 2.2Communication skills-I can participate in online group chats (community activity groups) via my phone. | 0.755 | 0.977 |  |
| I can use the internet to communicate with others—chatting with family on WeChat, sharing videos with friends on Douyin. | 0.795 | 0.976 |  |
| I can proficiently take photos and post them on Moments or Douyin. | 0.767 | 0.976 |  |
| 2.3Information Security and Privacy Protection Skills-I can set privacy protections like phone passwords to safeguard personal information. | 0.765 | 0.977 |  |
| I can recognize scam calls and texts and refuse to answer them. | 0.788 | 0.976 |  |
| I can protect my finances online—verifying recipient details when using mobile payments. | 0.785 | 0.976 |  |
| I can search for information and stay informed about current events via mobile internet platforms. | 0.749 | 0.977 |  |
| 2.4Problem-solving skills-When my phone lags, I clear memory and restart it to restore performance. | 0.781 | 0.976 |  |
| I resolve technical and information issues by calling customer service—e.g., recharging credits, checking data usage. | 0.773 | 0.976 |  |
| I learn new features by following manuals—e.g., QR code payments. | 0.769 | 0.976 |  |
| 2.5Information Interaction Skills-I complete QR code payments using WeChat/Alipay. | 0.766 | 0.976 |  |
| I book hospital appointments and make online payments via mobile apps. | 0.776 | 0.976 |  |
| I plan travel routes using map navigation software. | 0.786 | 0.976 |  |
| I proficiently use public services apps to pay utilities, social insurance, and check pension balances. | 0.795 | 0.976 |  |
| I can search for products, place orders, and process returns/exchanges on e-commerce platforms (e.g., Taobao). | 0.778 | 0.976 |  |
| 3.1Frequency of Use-I use my phone for over an hour daily (scrolling through Douyin, chatting, reading news). | 0.758 | 0.977 |  |
| I frequently use my phone and other digital devices weekly. | 0.779 | 0.976 |  |
| 3.2Versatility in usage scenarios-I've used my phone for: shopping, government services, and social networking. | 0.771 | 0.976 |  |
| I often use my phone to listen to music, watch operas, tune into the radio, and read novels. | 0.778 | 0.976 |  |
| I regularly use my phone for learning and acquiring new skills. | 0.744 | 0.977 |  |

As shown in table 1: The reliability coefficient for the mean digital competence score is 0.977. Regarding the "alpha coefficients for deleted items," the reliability coefficients after item deletion are all lower than the overall 0.977. Regarding the "CITC values," the CITC values for all analyzed items exceed 0.4, indicating strong correlations among the analyzed items and confirming a high level of reliability. In summary, the reliability coefficient of the research data is 0.977, comprehensively indicating excellent data reliability quality.

Table 2 **Methods for Measuring Subjective Well-Being**

| NAME | Correlation of Total Item Correction  (CITC) | α Coefficient of Deleted Items | Cronbach αCoefficient |
| --- | --- | --- | --- |
| Very satisfied with your life? | 0.793 | 0.968 | 0.970 |
| Very fortunate | 0.761 | 0.968 |  |
| Generally speaking, have your life circumstances become more satisfying? | 0.765 | 0.968 |  |
| Negative Affect (NA)。In recent months, you have felt: (The closer to the right, the higher the value)-Worries | 0.755 | 0.968 |  |
| Feeling very lonely or isolated | 0.757 | 0.968 |  |
| Feeling depressed or very unhappy | 0.750 | 0.968 |  |
| Worried about what the future holds | 0.744 | 0.968 |  |
| Feeling your life circumstances have become difficult | 0.742 | 0.968 |  |
| Positive Experience (PE)。In recent months, you have felt: (The closer to the left, the higher the value)I feel as happy as I did when I was young | 0.743 | 0.968 |  |
| The things I do interest me as much as they used to | 0.746 | 0.968 |  |
| Looking back on my life, I feel quite satisfied | 0.782 | 0.968 |  |
| If you could live anywhere you wanted, where would you choose to live? | 0.764 | 0.968 |  |
| Am I as happy now as I was when I was young? | 0.744 | 0.968 |  |
| Were you satisfied with your life back then? | 0.760 | 0.968 |  |
| My health is the same as or even better than that of my peers | 0.739 | 0.968 |  |
| Negative Experience (NE)。In recent months, you have felt: (The closer to the left, the higher the value)This is the most difficult period of my life | 0.757 | 0.968 |  |
| Most of what I do is tedious or monotonous | 0.749 | 0.968 |  |
| As I get older, everything gets worse. | 0.747 | 0.968 |  |
| Do you feel lonely? | 0.759 | 0.968 |  |
| Have some things troubled me this year? | 0.784 | 0.968 |  |
| Sometimes I feel life is meaningless. | 0.773 | 0.968 |  |
| Most of the time I feel life is hard | 0.734 | 0.968 |  |

As shown in table 2: The reliability coefficient for the mean subjective well-being score is 0.970. Regarding the "alpha coefficients for deleted items," the reliability coefficients after item deletion are all below the overall value of 0.970. Concerning the "CITC values," the CITC values for all analyzed items exceed 0.4, indicating strong correlations among the analyzed items and confirming a high level of reliability. In summary, the reliability coefficient of the research data is 0.970, collectively indicating extremely high data reliability quality.

Table 3 **Measurement Methods for Cultural Deprivation**

| Name | Correlation of Total Item Correction  (CITC) | α Coefficient of Deleted Items | CronbachαCoefficient |
| --- | --- | --- | --- |
| Online Cultural Deprivation-Video Category — I frequently watch short videos, opera performances, square dances, etc., on platforms like Douyin and Kuaishou. | 0.711 | 0.958 | 0.960 |
| Video Category — I can easily find long-form videos, TV series, and movies that interest me on Tencent Video/iQIYI/Youku/Bilibili. | 0.737 | 0.958 |  |
| Video Category — I often watch live events such as talent shows and intangible cultural heritage performances. | 0.737 | 0.958 |  |
| Audio Category — I regularly listen to audiobooks, radio dramas, and storytelling programs, such as those on Himalaya. | 0.700 | 0.958 |  |
| Reading Category — I frequently read e-books/digital novels | 0.766 | 0.957 |  |
| Access to Online Cultural Information-Knowledge Popularization Category — I acquire health/wellness knowledge, historical/cultural knowledge, and scientific knowledge through online lectures/open courses | 0.706 | 0.958 |  |
| Knowledge Popularization Category — I receive updates on intangible cultural heritage events and festival information via official websites/government portals/Ministry of Culture and Tourism/local cloud platforms | 0.741 | 0.958 |  |
| Practical Skills — I learn paper-cutting, weaving, and cooking tutorials through platforms like Douyin and Kuaishou | 0.721 | 0.958 |  |
| Practical Skills — I access illustrated guides for “mobile registration, ride-hailing, and advance appointments” via community official accounts, mini-programs, or Xiaohongshu | 0.753 | 0.957 |  |
| Barriers to Offline Smart Services-Venue Reservations & Guided Tours — I can independently complete online reservations for museums/libraries without assistance from children or volunteers | 0.707 | 0.958 |  |
| Venue Reservations & Guided Tours — I can independently operate AR maps at the Forbidden City and immersive exhibition equipment at memorial halls without staff guidance | 0.726 | 0.958 |  |
| Self-Service Facilities — I can independently use library self-checkout systems via facial recognition or QR code scanning | 0.736 | 0.958 |  |
| Self-Service Facilities — I can independently rent and return self-guided audio devices | 0.750 | 0.957 |  |
| Community Cultural Activities — I can independently register for community events via WeChat groups/mini-programs without needing children to set reminders | 0.748 | 0.957 |  |
| Community Cultural Activities — I can independently accumulate points for offline cultural activities via apps and redeem gifts | 0.736 | 0.958 |  |
| Perception of Cultural Welfare-Participating in cultural activities brings me joy | 0.701 | 0.958 |  |
| I've made new friends through cultural activities | 0.761 | 0.957 |  |
| Current cultural resources meet my spiritual needs | 0.737 | 0.958 |  |
| Smart services have enhanced the convenience of my cultural participation | 0.705 | 0.958 |  |

As shown in table 3: The reliability coefficient for the mean cultural welfare score is 0.960. Regarding the "alpha coefficients for deleted items," the reliability coefficients after item deletion are all below the overall value of 0.960. Regarding the "CITC values," the CITC values for all analyzed items exceed 0.4, indicating strong correlations among the analyzed items and confirming a high level of reliability. In summary, the reliability coefficient of the research data is 0.960, comprehensively indicating excellent data reliability quality.

## Reliability Statistics

Table 4 **Reliability Statistics**

| Reliability Statistics | | |
| --- | --- | --- |
| Dimension Name | Cronbach Alpha | Number of Items |
| Average Digital Competence | 0.977 | 27 |
| Average Subjective Well-being | 0.970 | 22 |
| Average Cultural Welfare | 0.960 | 19 |

## Overall Reliability Analysis

Table 5 **Simplified Format for Reliability**

| Cronbach Alpha | Sample size | Number of items |
| --- | --- | --- |
| 0.967 | 500 | 68 |

As can be seen from the overall reliability coefficient, the standardized reliability coefficient is 0.967, indicating that the questionnaire as a whole exhibits excellent reliability.

## Validity Analysis

Table 6 **KMO and Bartlett's Test**

| KMO Sampling Adequacy Measure | | 0.977 |
| --- | --- | --- |
| Bartlett's Sphericity Test | Approximate Chi-Square | 26712.623 |
|  | Degrees of Freedom | 2278.000 |
|  | Significance | 0.000 |

Validity was verified using the KMO and Bartlett tests. The KMO test yielded a coefficient of 0.977, while the Bartlett test produced a chi-square value of 26,712.623 (Sig. = 0.000 < 0.01). These results indicate that the questionnaire exhibits excellent overall validity.

Table 7 **Variance Explained Table**

| Factor Number | Characteristic Root | | | Rotation Forward Difference Interpretation Rate | | | | Rotation Backward Difference Interpretation Rate | | | |
| --- | --- | --- | --- | --- | --- | --- | --- | --- | --- | --- | --- |
|  | Total | Percentage of variance explained | Cumulative % | Total | Percentage of variance explained | Cumulative % | Total | | Percentage of variance explained | Cumulative % |  |
| 1 | 22.232 | 32.695 | 32.695 | 22.232 | 32.695 | 32.695 | 16.957 | | 24.937 | 24.937 |  |
| 2 | 11.055 | 16.257 | 48.952 | 11.055 | 16.257 | 48.952 | 13.484 | | 19.829 | 44.765 |  |
| 3 | 8.292 | 12.195 | 61.146 | 8.292 | 12.195 | 61.146 | 11.139 | | 16.381 | 61.146 |  |
| 4 | 0.760 | 1.118 | 62.264 |  |  |  |  | |  |  |  |
| 5 | 0.742 | 1.091 | 63.355 |  |  |  |  | |  |  |  |
| 6 | 0.718 | 1.056 | 64.410 |  |  |  |  | |  |  |  |
| 7 | 0.706 | 1.038 | 65.449 |  |  |  |  | |  |  |  |
| 8 | 0.685 | 1.007 | 66.456 |  |  |  |  | |  |  |  |
| 9 | 0.666 | 0.979 | 67.435 |  |  |  |  | |  |  |  |
| 10 | 0.654 | 0.962 | 68.397 |  |  |  |  | |  |  |  |
| 11 | 0.634 | 0.933 | 69.330 |  |  |  |  | |  |  |  |
| 12 | 0.611 | 0.898 | 70.228 |  |  |  |  | |  |  |  |
| 13 | 0.602 | 0.885 | 71.113 |  |  |  |  | |  |  |  |
| 14 | 0.590 | 0.867 | 71.980 |  |  |  |  | |  |  |  |
| 15 | 0.576 | 0.847 | 72.827 |  |  |  |  | |  |  |  |
| 16 | 0.563 | 0.828 | 73.656 |  |  |  |  | |  |  |  |
| 17 | 0.557 | 0.820 | 74.475 |  |  |  |  | |  |  |  |
| 18 | 0.537 | 0.790 | 75.265 |  |  |  |  | |  |  |  |
| 19 | 0.533 | 0.784 | 76.049 |  |  |  |  | |  |  |  |
| 20 | 0.513 | 0.754 | 76.804 |  |  |  |  | |  |  |  |
| 21 | 0.509 | 0.748 | 77.552 |  |  |  |  | |  |  |  |
| 22 | 0.502 | 0.738 | 78.289 |  |  |  |  | |  |  |  |
| 23 | 0.490 | 0.721 | 79.010 |  |  |  |  | |  |  |  |
| 24 | 0.486 | 0.715 | 79.726 |  |  |  |  | |  |  |  |
| 25 | 0.471 | 0.693 | 80.419 |  |  |  |  | |  |  |  |
| 26 | 0.464 | 0.683 | 81.101 |  |  |  |  | |  |  |  |
| 27 | 0.458 | 0.674 | 81.776 |  |  |  |  | |  |  |  |
| 28 | 0.446 | 0.655 | 82.431 |  |  |  |  | |  |  |  |
| 29 | 0.442 | 0.650 | 83.081 |  |  |  |  | |  |  |  |
| 30 | 0.433 | 0.637 | 83.718 |  |  |  |  | |  |  |  |
| 31 | 0.419 | 0.616 | 84.334 |  |  |  |  | |  |  |  |
| 32 | 0.413 | 0.608 | 84.942 |  |  |  |  | |  |  |  |
| 33 | 0.401 | 0.590 | 85.532 |  |  |  |  | |  |  |  |
| 34 | 0.396 | 0.583 | 86.115 |  |  |  |  | |  |  |  |
| 35 | 0.386 | 0.567 | 86.682 |  |  |  |  | |  |  |  |
| 36 | 0.381 | 0.561 | 87.243 |  |  |  |  | |  |  |  |
| 37 | 0.376 | 0.553 | 87.796 |  |  |  |  | |  |  |  |
| 38 | 0.365 | 0.537 | 88.334 |  |  |  |  | |  |  |  |
| 39 | 0.362 | 0.532 | 88.866 |  |  |  |  | |  |  |  |
| 40 | 0.361 | 0.530 | 89.396 |  |  |  |  | |  |  |  |
| 41 | 0.346 | 0.509 | 89.905 |  |  |  |  | |  |  |  |
| 42 | 0.337 | 0.495 | 90.401 |  |  |  |  | |  |  |  |
| 43 | 0.334 | 0.492 | 90.892 |  |  |  |  | |  |  |  |
| 44 | 0.327 | 0.481 | 91.374 |  |  |  |  | |  |  |  |
| 45 | 0.322 | 0.474 | 91.847 |  |  |  |  | |  |  |  |
| 46 | 0.318 | 0.468 | 92.315 |  |  |  |  | |  |  |  |
| 47 | 0.311 | 0.458 | 92.772 |  |  |  |  | |  |  |  |
| 48 | 0.296 | 0.436 | 93.208 |  |  |  |  | |  |  |  |
| 49 | 0.295 | 0.434 | 93.642 |  |  |  |  | |  |  |  |
| 50 | 0.283 | 0.417 | 94.059 |  |  |  |  | |  |  |  |
| 51 | 0.278 | 0.409 | 94.467 |  |  |  |  | |  |  |  |
| 52 | 0.268 | 0.394 | 94.862 |  |  |  |  | |  |  |  |
| 53 | 0.266 | 0.391 | 95.253 |  |  |  |  | |  |  |  |
| 54 | 0.264 | 0.388 | 95.641 |  |  |  |  | |  |  |  |
| 55 | 0.261 | 0.384 | 96.025 |  |  |  |  | |  |  |  |
| 56 | 0.250 | 0.368 | 96.393 |  |  |  |  | |  |  |  |
| 57 | 0.235 | 0.346 | 96.740 |  |  |  |  | |  |  |  |
| 58 | 0.233 | 0.343 | 97.083 |  |  |  |  | |  |  |  |
| 59 | 0.232 | 0.341 | 97.423 |  |  |  |  | |  |  |  |
| 60 | 0.230 | 0.338 | 97.762 |  |  |  |  | |  |  |  |
| 61 | 0.218 | 0.320 | 98.082 |  |  |  |  | |  |  |  |
| 62 | 0.208 | 0.306 | 98.388 |  |  |  |  | |  |  |  |
| 63 | 0.205 | 0.301 | 98.689 |  |  |  |  | |  |  |  |
| 64 | 0.194 | 0.285 | 98.974 |  |  |  |  | |  |  |  |
| 65 | 0.188 | 0.277 | 99.251 |  |  |  |  | |  |  |  |
| 66 | 0.178 | 0.262 | 99.513 |  |  |  |  | |  |  |  |
| 67 | 0.172 | 0.253 | 99.766 |  |  |  |  | |  |  |  |
| 68 | 0.159 | 0.234 | 100.000 |  |  |  |  | |  |  |  |

Extraction method: Principal Component Analysis.

The table above presents the factor extraction results and the information content of each extracted factor. As shown in table 7, factor analysis extracted a total of three factors, all with eigenvalues greater than 1. After rotation, these three factors explained 24.937%, 19.829%, and 16.381% of the variance, respectively, with a cumulative variance explained of 61.146%.

Table 8 Rotation-Adjusted Factor Loadings Table

| Name | Factor loading coefficient | | | Shared Experience(Common factor variance) |
| --- | --- | --- | --- | --- |
|  | factor1 | factor2 | factor3 |  |
| 1.1 Equipment Accessibility—I have a smartphone that can access the internet and functions normally. | 0.772 | 0.200 | 0.077 | 0.642 |
| I can afford the monthly phone bill and data plan. | 0.766 | 0.142 | 0.037 | 0.608 |
| 1.2 Network Coverage—My phone has excellent network coverage, allowing me to browse news and watch videos smoothly. | 0.772 | 0.137 | 0.009 | 0.614 |
| My data plan's allowance and duration fully meet my daily usage needs. | 0.797 | 0.175 | 0.052 | 0.668 |
| 2.1 Basic operational capabilities (physical contact and simple interaction)—I can independently use a smartphone to make and receive calls. | 0.792 | 0.070 | 0.010 | 0.632 |
| I am proficient in sending and receiving text messages or WeChat voice messages. | 0.791 | 0.171 | 0.038 | 0.656 |
| I can adjust basic settings such as phone volume and brightness. | 0.789 | 0.151 | 0.017 | 0.646 |
| 2.2 Communication Skills—I can participate in online group discussions via my mobile phone.（Community Activity Group） | 0.773 | 0.094 | -0.022 | 0.607 |
| I can use the internet to communicate with others—chatting with family on WeChat and sharing videos with friends on TikTok. | 0.790 | 0.195 | 0.011 | 0.663 |
| I am proficient in taking photos and posting them on social media platforms like WeChat Moments and TikTok. | 0.775 | 0.119 | 0.064 | 0.619 |
| 2.3Information Security and Privacy Protection Capabilities—I can set privacy settings such as a phone passcode to protect your personal privacy. | 0.769 | 0.142 | 0.052 | 0.615 |
| I can identify scam calls and scam texts and reject them | 0.792 | 0.134 | 0.064 | 0.650 |
| I can protect my assets online.——Use mobile payment to verify the recipient's information | 0.774 | 0.213 | 0.041 | 0.647 |
| I can search for information and stay informed about current events through mobile internet platforms. | 0.760 | 0.109 | 0.071 | 0.594 |
| 2.4 Problem-solving ability—When my phone lags, I clear the memory and restart it to recover. | 0.780 | 0.166 | 0.059 | 0.639 |
| I will resolve technical and information issues by calling customer service.——For example, top-up spending, data usage inquiry | 0.773 | 0.170 | 0.024 | 0.627 |
| I well learn new features by following the manual—such as scanning QR codes for payment. | 0.780 | 0.104 | 0.070 | 0.623 |
| 2.5Information exchange capability(Information Retrieval and Online Services)—I can use WeChat Pay or Alipay to complete the QR code payment. | 0.768 | 0.153 | 0.046 | 0.615 |
| I can book hospital appointments and make online payments through a mobile app. | 0.782 | 0.146 | -0.001 | 0.633 |
| I can use map navigation software to plan my travel route. | 0.782 | 0.178 | 0.048 | 0.646 |
| I am proficient in using daily life and government services, including paying utility bills, social security contributions, and checking pension balances. | 0.795 | 0.174 | 0.000 | 0.662 |
| I can complete product searches, place orders, and process returns and exchanges on e-commerce platforms such as Taobao. | 0.779 | 0.146 | 0.093 | 0.637 |
| 3.1Frequency of Use—I use my phone for over an hour every day (scrolling through tiktok, chatting, reading news). | 0.767 | 0.117 | 0.059 | 0.606 |
| I use digital devices such as my phone frequently every week. | 0.788 | 0.110 | 0.075 | 0.639 |
| 3.2Versatility in usage scenarios—I've used my phone to accomplish the following: shopping, government services, and social networking. | 0.771 | 0.173 | 0.011 | 0.624 |
| I often use my phone to listen to music, watch operas, tune in to radio broadcasts, and read novels. | 0.780 | 0.163 | 0.017 | 0.635 |
| I often use my phone to learn new skills. | 0.741 | 0.176 | 0.077 | 0.585 |
| Are you satisfied with your life? | 0.110 | 0.807 | 0.073 | 0.669 |
| Very fortunate | 0.135 | 0.771 | 0.074 | 0.618 |
| Generally speaking, have your living conditions become satisfactory? | 0.151 | 0.775 | 0.045 | 0.625 |
| Negative Affect(NA)：In recent months, you've been feeling a bit troubled.：（Values to the right are larger.） | 0.161 | 0.763 | 0.053 | 0.610 |
| Extremely lonely or estranged from others | 0.132 | 0.763 | 0.099 | 0.610 |
| Depressed or extremely unhappy | 0.111 | 0.763 | 0.095 | 0.604 |
| Worried because I don't know what will happen. | 0.187 | 0.735 | 0.138 | 0.594 |
| feel that your life has become difficult | 0.116 | 0.745 | 0.162 | 0.595 |
| Positive Experience(PE)：In recent months, you've felt as happy as you did in your youth.（Values shifted to the left are larger values.） | 0.149 | 0.744 | 0.121 | 0.591 |
| What I do still interests me as much as before. | 0.135 | 0.761 | 0.039 | 0.598 |
| When I look back on my life, I feel quite satisfied. | 0.148 | 0.786 | 0.092 | 0.648 |
| If you could live anywhere you wanted, where would you choose to live? | 0.139 | 0.764 | 0.142 | 0.623 |
| I'm as happy now as I was in my youth.？ | 0.173 | 0.744 | 0.080 | 0.590 |
| Are you satisfied with your life back then?？ | 0.151 | 0.766 | 0.074 | 0.616 |
| My health is comparable to that of my peers, and in some ways even better. | 0.188 | 0.729 | 0.141 | 0.586 |
| Negative Experience(NE)：The past few months have been the most difficult period of my life.（Values to the right are larger.） | 0.217 | 0.744 | 0.119 | 0.615 |
| Most of what I do is tedious or monotonous. | 0.127 | 0.759 | 0.091 | 0.601 |
| Do you think everything gets worse as you get older? | 0.210 | 0.744 | 0.044 | 0.600 |
| Do you feel lonely?？ | 0.161 | 0.764 | 0.069 | 0.614 |
| This year, certain things have been troubling me.？ | 0.185 | 0.771 | 0.162 | 0.655 |
| Sometimes I feel like life has no meaning.？ | 0.210 | 0.768 | 0.052 | 0.637 |
| Most of the time, I find life to be tough. | 0.190 | 0.737 | 0.043 | 0.581 |
| Online Cultural Service Participation—Video Category——I often watch short videos, traditional opera, and square dancing on platforms like tiktok | 0.010 | 0.081 | 0.739 | 0.553 |
| Video Category——I can easily find long-form videos, TV series, and movies that interest me on Tencent Video, iQIYI, Youku, and Bilibili. | 0.020 | 0.103 | 0.761 | 0.591 |
| Video Category——I often watch live events such as talent shows and intangible cultural heritage performances. | 0.023 | 0.096 | 0.761 | 0.589 |
| Audio Category — I regularly listen to audiobooks, radio dramas, and storytelling programs, such as those on Himalaya. | -0.011 | 0.055 | 0.735 | 0.543 |
| Reading Category — I frequently read e-books/digital novels | 0.044 | 0.094 | 0.789 | 0.633 |
| Knowledge Popularization Category — I acquire health/wellness knowledge, historical/cultural knowledge, and scientific knowledge through online lectures/open courses | 0.052 | 0.064 | 0.735 | 0.546 |
| Knowledge Popularization Category — I receive updates on intangible cultural heritage events and festival information via official websites/government portals/Ministry of Culture and Tourism/local cloud platforms | 0.052 | 0.095 | 0.765 | 0.596 |
| Art Appreciation Category — I appreciate cultural relics and landscapes through virtual exhibitions (museum cloud platforms, online Dunhuang tours, VR platforms) | 0.030 | 0.042 | 0.753 | 0.569 |
| Art Appreciation——I watched the online performance via the National Centre for the Performing Arts' “Cloud Theater” and live streams from local opera troupes. | 0.053 | 0.138 | 0.770 | 0.615 |
| Offline Smart Service Barriers—Venue Reservations and Guided Tours—I can independently complete online reservations for museums/libraries without needing assistance from my children or volunteers. | 0.073 | 0.103 | 0.729 | 0.548 |
| Venue Reservations and Guided Tours — I can independently operate the equipment for the Forbidden City AR map and immersive exhibitions at the memorial hall without staff assistance. | 0.091 | 0.106 | 0.747 | 0.577 |
| Self-service facilities—I can independently complete library self-checkout using facial recognition or QR code scanning. | 0.046 | 0.064 | 0.764 | 0.589 |
| Self-service facilities—I can independently complete the rental and return of self-guided audio devices. | -0.000 | 0.071 | 0.778 | 0.611 |
| Community Cultural Activities — I can independently sign up for community events via the neighborhood WeChat group or mini-program without needing my children to set reminders for me. | -0.011 | 0.108 | 0.773 | 0.608 |
| Community Cultural Activities — I can independently earn points for offline cultural activities within the app and redeem them for gifts. | 0.052 | 0.111 | 0.758 | 0.589 |
| Cultural Well-being Perception—Participating in cultural activities brings me joy. | 0.069 | 0.109 | 0.723 | 0.539 |
| I made new friends through cultural activities. | 0.033 | 0.006 | 0.795 | 0.633 |
| The existing cultural resources can satisfy my spiritual needs. | 0.080 | 0.130 | 0.754 | 0.592 |
| Smart services have enhanced the convenience of my cultural participation. | 0.065 | 0.089 | 0.729 | 0.544 |

Extraction Method: Principal Component Analysis.

Rotation Method: Kaiser Normalized Maximum Variance Method.

Note: Colored values in the table indicate:

Green represents load factor absolute values greater than 0.4.

The data in this study were rotated using the Varimax method to identify the correspondence between factors and research items. The table above presents the information extraction results of factors for each research item, as well as the correspondence between factors and items. As shown in table 8: the communality values for all research items are above 0.4, indicating a strong association between the items and factors, and that the factors can effectively extract relevant information. After confirming that the factors can capture most of the information from the research items, the next step is to analyze the correspondence between factors and items (an absolute factor loading coefficient greater than 0.4 denotes a significant corresponding relationship between the item and the factor).


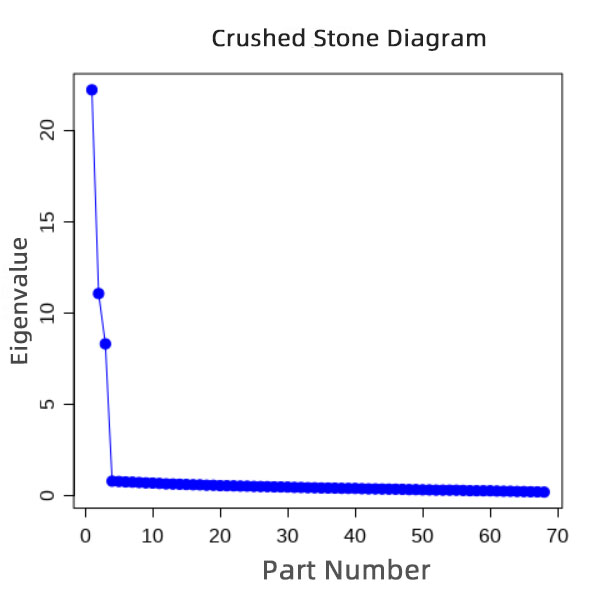


Figure 1 Crushed Stone Diagram

The scree plot is a graph that illustrates how well each principal component explains the variation in the data. Its purpose is to determine the number of principal components to select based on the rate of decrease in eigenvalues. Combined with the figure 1, it can be used to confirm or adjust the number of principal components. Each principal component is represented as a point. The number of principal components to extract is determined by identifying the point where the slope begins to flatten. (For example, if the data flattens after the third principal component, then the first three principal components can be extracted.)

1. **Empirical Analysis**

3.1 **Descriptive Analysis**

Table 9 Descriptive Statistics of Variables (N=500)

| Descriptive Analysis |  |  |  |  |  |
| --- | --- | --- | --- | --- | --- |
|  | N | Minimum | Maximum | Mean | Standard Deviation |
| Dependent Variable |  |  |  |  |  |
| Subjective Well-Being | 500 | .00 | 96.00 | 66.4040 | 28.04964 |
| Independent Variable |  |  |  |  |  |
| Digital Disability | 500 | 45.00 | 134.00 | 100.0160 | 25.00124 |
| Moderating Variable |  |  |  |  |  |
| Cultural Deprivation | 500 | 20.00 | 95.00 | 58.4200 | 16.03514 |
| Control Variables |  |  |  |  |  |
| Age (1=50-55) | 500 | 1 | 6 | 2.94 | 1.400 |
| Gender (1=Male) | 500 | 1 | 2 | 1.53 | .500 |
| Education Level (1=No Formal Education) | 500 | 1 | 6 | 4.06 | 1.211 |
| Residence (1=Rural) | 500 | 1 | 4 | 1.87 | .885 |
| Source of Income (1=Pension) | 500 | 1 | 5 | 3.01 | 1.138 |
| Health Status (1=Fully Self-Sufficient) | 500 | 1 | 3 | 1.30 | .578 |
| Relationship with Children (1=Close) | 500 | 1 | 3 | 1.35 | .641 |
| Occupation (1=Farmer) | 500 | 1 | 7 | 2.27 | 1.848 |
| Number of Valid Cases (Per Row) | 500 |  |  |  |  |

1. Analysis of Core Research Variables

As shown in table 9 the scores for the three core variables are as follows:The scores for the three core variables are as follows:

Subjective Well-being: The mean score was 66.40 (out of 96), indicating an above-average level. However, the large standard deviation (28.05) and wide range from 0 to 96 suggest highly significant individual differences in well-being among the elderly sample, encompassing both extremely happy and profoundly unhappy individuals.

Digital Competence: The average score was 100.02 (out of 135), indicating the sample's overall digital competence is at a moderate level. The large standard deviation (25.00) and wide score range (45-134) similarly reveal a substantial digital divide within the elderly population, reflecting significant disparities in digital skill proficiency among individuals.

Cultural Welfare: The average score was 58.42 (out of 95), the lowest among the three core variables. Its standard deviation (16.04) also indicates pronounced inequality in older adults' access to and enjoyment of cultural welfare.

2. Analysis of Sample Demographic Characteristics

The mean values and coding of demographic variables reveal the sample's composition:

Age: The mean was 2.94 (between codes 2 and 3). Combined with frequency analysis (see main report), the sample predominantly consists of “younger seniors” aged 56-65, with a lower proportion of older seniors (e.g., aged 76 and above).

Gender: The mean is 1.53 (1=male, 2=female), indicating a slightly higher proportion of females (approximately 53%) in the sample. This aligns with the higher proportion of women in China's aging population.

Education Level: The mean score is 4.06 (between codes 3 and 4). Frequency analysis indicates that the sample's educational background is predominantly junior high school and senior high school/vocational school, reflecting an overall intermediate level of education.

Residential Status: The mean score is 1.87, indicating that the sample primarily consists of elderly individuals residing in rural areas and urban communities, consistent with the predominant residential patterns of China's elderly population.

Source of Income: The average score is 3.01. Based on the coding, the elderly's income sources fall between “government subsidies” and “labor income,” reflecting diverse livelihood patterns.

Health Status: The mean value of 1.30 heavily leans toward Code 1 (Fully Self-Sufficient), indicating that the vast majority of elderly individuals in this sample are in good health and primarily capable of self-care.

Relationship with Children: The mean value of 1.35 is very close to Code 1 (Close), suggesting that the elderly in the sample generally perceive their relationships with their children as relatively close.

Occupation: The mean value is 2.27. Combined with frequency analysis, it is evident that farmers constitute the largest occupational group in the sample.

3. Data Quality Assessment

All 500 case data sets are complete with no missing values, providing a high-quality data foundation for subsequent statistical analysis. The distribution ranges of each variable are sufficiently broad, with no instances of extreme compression, making the data suitable for further parametric testing and model construction.

**3.2 Correlation Analysis**

Table 10 Pearson Correlation Analysis

|  | Mean | Standard Deviation |  | Subjective well-being | digital competence | cultural welfare |
| --- | --- | --- | --- | --- | --- | --- |
| Subjective well-being | 66.404 | 28.050 | Pearson correlation | 1 |  |  |
|  |  |  | Sig. (Double-tailed) |  |  |  |
| digital competence | 100.016 | 25.001 | Pearson correlation | 0.389*** | 1 |  |
|  |  |  | Sig. (Double-tailed) | 0.000 |  |  |
| cultural welfare | 58.420 | 16.035 | Pearson correlation | 0.235*** | 0.125** | 1 |
|  |  |  | Sig. (Double-tailed) | 0.000 | 0.005 |  |

*** Significant at the 0.001 level (two-tailed).

** Significant at the 0.01 level (two-tailed).

* Significant at the 0.05 level (two-tailed).

To preliminarily validate the research hypotheses, this study conducted Pearson correlation analyses， as shown in table 10 Results confirmed that both digital competence (r = 0.389, p < 0.001) and cultural welfare (r = 0.235, p < 0.001) exhibited significant positive correlations with subjective well-being, providing preliminary support for hypotheses H1 and H2. Additionally, a significant positive correlation was found between digital competence and cultural well-being (r = 0.125, p < 0.01), indicating that while these are distinct constructs, they exhibit a degree of synergy.

**3.3**  **Linear Regression Analysis**

Table 11 Linear Regression Analysis Results for Subjective Well-Being (n=500)

|  | Non-standardization coefficient | Standardized Coefficient | | t | p | LLCI | ULCI | VIF |
| --- | --- | --- | --- | --- | --- | --- | --- | --- |
|  | B | Standard error | Beta |  |  |  |  |  |
| Constant | 19.134 | 11.070 |  | 1.728 | 0.085 | -2.616 | 40.885 |  |
| Age | -0.842 | 1.053 | -0.042 | -0.800 | 0.424 | -2.911 | 1.227 | 1.626 |
| Gender | -0.693 | 2.350 | -0.012 | -0.295 | 0.768 | -5.310 | 3.923 | 1.031 |
| Educational background | 1.208 | 1.174 | 0.052 | 1.029 | 0.304 | -1.098 | 3.515 | 1.513 |
| Housing Situation | -1.495 | 1.341 | -0.047 | -1.115 | 0.265 | -4.128 | 1.139 | 1.054 |
| Source of income | 0.069 | 1.082 | 0.003 | 0.064 | 0.949 | -2.057 | 2.195 | 1.134 |
| Health Status | 0.140 | 2.031 | 0.003 | 0.069 | 0.945 | -3.851 | 4.132 | 1.031 |
| Parent-Child Relationship | 4.254 | 1.817 | 0.097 | 2.341 | 0.020 | 0.684 | 7.824 | 1.015 |
| Occupation | -0.286 | 0.669 | -0.019 | -0.427 | 0.670 | -1.600 | 1.029 | 1.143 |
| digital competence | 0.432 | 0.046 | 0.385 | 9.296 | 0.000 | 0.341 | 0.524 | 1.011 |
| R-squared | 0.167 | | | | | | | |
| Adjust R-squared | 0.152 | | | | | | | |
| F | F(9,500)=10.953,p=0.000 | | | | | | | |
| D-W | 1.936 | | | | | | | |
| Dependent variable | Subjective well-being | | | | | | | |

To further clarify the causal relationships among variables, this study established a linear regression model with subjective well-being as the dependent variable, controlling for demographic variables such as age, gender, and Educational background(see table 11). The regression analysis revealed that the model was significantly significant overall (F = 10.953, p < 0.001), with an adjusted R² of 0.152. Among the numerous independent variables, digital competence (β = 0.385, p < 0.001) and parent-child relationships (β = 0.097, p < 0.05) demonstrated significant positive predictive effects on subjective well-being, while the influence of other control variables failed to reach statistical significance. This finding further reinforces the assertion that digital literacy is a key factor in enhancing older adults' well-being.

**3.4 Moderation Effect Analysis**

Table 12 Stratified Regression Analysis Results for the Moderating Effect of Cultural Welfare

|  | Class 1 Regression (X => Y)Subjective well-being | | | | Class 2 Regression (X+W => Y)Subjective well-being | | | | Class 3 Regression(X+W+XW=>Y)Subjective well-being | | | |
| --- | --- | --- | --- | --- | --- | --- | --- | --- | --- | --- | --- | --- |
|  | B | Standard error | t | p | B | Standard error | t | p | B | Standard error | t | p |
| Constant | 62.367 | 10.059 | 6.200 | 0.000 | 59.033 | 9.883 | 5.973 | 0.000 | 58.736 | 9.804 | 5.991 | 0.000 |
| Age | -0.842 | 1.053 | -0.800 | 0.424 | -0.560 | 1.034 | -0.541 | 0.588 | -0.568 | 1.025 | -0.554 | 0.580 |
| Gender | -0.693 | 2.350 | -0.295 | 0.768 | -0.448 | 2.303 | -0.195 | 0.846 | -0.779 | 2.287 | -0.341 | 0.734 |
| Educational background | 1.208 | 1.174 | 1.029 | 0.304 | 1.121 | 1.150 | 0.975 | 0.330 | 1.326 | 1.143 | 1.160 | 0.246 |
| Housing Situation | -1.495 | 1.341 | -1.115 | 0.265 | -1.479 | 1.314 | -1.126 | 0.261 | -1.622 | 1.304 | -1.244 | 0.214 |
| Source of income | 0.069 | 1.082 | 0.064 | 0.949 | 0.543 | 1.065 | 0.510 | 0.610 | 0.427 | 1.057 | 0.404 | 0.687 |
| Health Status | 0.140 | 2.031 | 0.069 | 0.945 | 0.472 | 1.992 | 0.237 | 0.813 | 0.693 | 1.977 | 0.351 | 0.726 |
| Parent-Child Relationship | 4.254 | 1.817 | 2.341 | 0.020 | 4.394 | 1.781 | 2.468 | 0.014 | 4.991 | 1.777 | 2.808 | 0.005 |
| Occupation | -0.286 | 0.669 | -0.427 | 0.670 | -0.107 | 0.657 | -0.162 | 0.871 | -0.142 | 0.652 | -0.218 | 0.828 |
| cultural welfare | 0.432 | 0.046 | 9.296 | 0.000 | 0.406 | 0.046 | 8.832 | 0.000 | 0.383 | 0.046 | 8.304 | 0.000 |
| cultural welfare |  |  |  |  | 0.331 | 0.072 | 4.617 | 0.000 | 0.246 | 0.077 | 3.213 | 0.001 |
| digital competence |  |  |  |  |  |  |  |  | -0.008 | 0.003 | -3.009 | 0.003 |
| R2 | 0.167 | | | | 0.202 | | | | 0.217 | | | |
| Adjust R2 | 0.152 | | | | 0.186 | | | | 0.199 | | | |
| F | F(9,500)=10.953,p=0.000 | | | | F(10,500)=12.397,p=0.000 | | | | F(11,500)=12.279,p=0.000 | | | |
| DW | 1.936 | | | | 1.944 | | | | 1.944 | | | |
|  | | | | | | | | | | | | |

The core objective of this study is to examine the moderating role of cultural deprivation in the relationship between digital competence and subjective well-being. Following the moderation effect testing procedure, we constructed a hierarchical regression model incorporating an interaction term, as shown in table 12.

Analysis revealed that introducing the interaction term between digital competence and cultural welfare significantly enhanced the model's explanatory power (ΔR² = 0.013, p < 0.01). The interaction term exhibited a negative and statistically significant regression coefficient (β = -0.008, p < 0.01), indicating that cultural welfare exerted a significant negative moderating effect on the relationship between digital literacy and well-being. Thus, research hypothesis H4 holds.

**3.4.1 Simple Slope Analysis**

Table 13 Results of Simple Slope Analysis for the Moderating Effect of Cultural Welfare

| Adjust variable levels | Regression coefficient | Standard error | t | p | LLCI | ULCI |
| --- | --- | --- | --- | --- | --- | --- |
| High Level (1SD) | 0.255 | 0.068 | 3.774 | 0.000 | 0.122 | 0.388 |
| Mean | 0.383 | 0.046 | 8.304 | 0.000 | 0.293 | 0.474 |
| Low Level (-1SD) | 0.511 | 0.058 | 8.889 | 0.000 | 0.398 | 0.624 |
| Note: LLCI denotes the lower bound of the 95% confidence interval for the estimate, while ULCI denotes the upper bound of the 95% confidence interval for the estimate. | | | | | | |

To further elucidate the specific form of this moderating effect, we conducted a simple slope analysis. As shown in table 13, under low cultural welfare levels (high deprivation conditions), the promotional effect of digital literacy on well-being was most pronounced (B = 0.511, p < 0.001); whereas at high cultural welfare levels (low deprivation context), this positive impact persists but significantly diminishes in strength (B = 0.255, p < 0.001). This finding strongly supports the “compensation effect” theory: when older adults face disadvantages in accessing traditional cultural welfare, enhanced digital literacy effectively compensates for their spiritual and cultural needs, thereby generating a stronger boost to well-being.


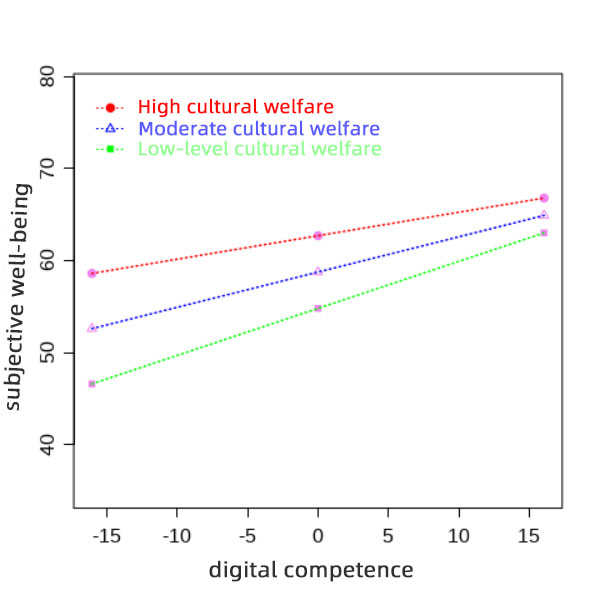


Figure 2 **Simple Slope Diagram of the Moderating Effect of Cultural Benefits on the Relationship Between Digital Competence and Subjective Well-Being**

The simple slope simple(see figure 2). slope plots reveal a clear moderation trend, with all slopes reaching statistical significance:

At low levels of cultural welfare (high cultural deprivation), digital competence exerts the strongest positive effect on well-being (B = 0.511).

At high levels of cultural welfare (low cultural deprivation), the positive effect of digital competence on well-being diminishes (B = 0.255).

This further strongly validates the “compensation effect” and “redundancy effect” proposed in the original text—namely, that cultural deprivation negatively moderates the relationship between digital competence and well-being. Hypothesis H4 is thus strongly supported once again.

**4.Hypothesis Test Results**

Based on theoretical analysis, this paper proposes the following research hypotheses. Through empirical analysis, these hypotheses are tested, with the specific results asas shown in table 14:

Table 14 Hypothesis Test Results

| Assume | | Assumed content | Result | |
| --- | --- | --- | --- | --- |
| H1 | Subjective well-being is positively correlated with cultural welfare. | | | Establishment |
| H2 | Subjective well-being is positively correlated with digital competence. | | | Establishment |
| H3 | Subjective well-being is positively correlated with both digital competence and cultural welfare. | | | Establishment |
| H4 | Cultural deprivation moderates the relationship between digital competence and subjective well-being. Specifically, when cultural deprivation is high, digital competence positively enhances well-being; whereas when cultural deprivation is low, this positive effect diminishes. | | | Establishment |

**5.Supplementary Summary of Phase II Data**

Based on the Phase II data analysis of the total sample of 500 participants, the primary conclusions from the initial study were comprehensively validated and reinforced:

1. Core variable relationships remain stable: The positive correlation between digital competence, cultural welfare, and subjective well-being remains statistically significant in the larger sample.

2. Moderating effects are significant with a clear pattern: The negative moderating effect of cultural welfare was confirmed, and simple slope analysis revealed a distinct operational pattern, providing a more robust empirical foundation for the original theoretical interpretation.

3. Enhanced research robustness: The addition of Phase II data significantly boosts the credibility and generalizability of findings, making the conclusions more reliable.。
